# Physiological Shear Stress Enhances Differentiation, Mucus-Formation and Structural 3D Organization of Intestinal Epithelial Cells In Vitro

**DOI:** 10.3390/cells10082062

**Published:** 2021-08-12

**Authors:** Marcus Lindner, Anna Laporte, Stephan Block, Laura Elomaa, Marie Weinhart

**Affiliations:** 1 Institute of Chemistry and Biochemistry, Freie Universität Berlin, 14195 Berlin, Germany; m.lindner@fu-berlin.de (M.L.); stephan.block@fu-berlin.de (S.B.); laura.elomaa@fu-berlin.de (L.E.); 2 Institute of Physical Chemistry and Electrochemistry, Leibniz Universität Hannover, 30167 Hannover, Germany; anna.laporte@pci.uni-hannover.de

**Keywords:** 3D-printed insert chamber, bioreactor, cell-based mucus model, cellular self-organization, CFD simulation, goblet cell differentiation, native mucus thickness, physiological fluid flow, reverse cell culture

## Abstract

Gastrointestinal (GI) mucus plays a pivotal role in the tissue homoeostasis and functionality of the gut. However, due to the shortage of affordable, realistic in vitro GI models with a physiologically relevant mucus layer, studies with deeper insights into structural and compositional changes upon chemical or physical manipulation of the system are rare. To obtain an improved mucus-containing cell model, we developed easy-to-use, reusable culture chambers that facilitated the application of GI shear stresses (0.002–0.08 dyn∙cm^−2^) to cells on solid surfaces or membranes of cell culture inserts in bioreactor systems, thus making them readily accessible for subsequent analyses, e.g., by confocal microscopy or transepithelial electrical resistance (TEER) measurement. The human mucus-producing epithelial HT29-MTX cell-line exhibited superior reorganization into 3-dimensional villi-like structures with highly proliferative tips under dynamic culture conditions when compared to static culture (up to 180 vs. 80 µm in height). Additionally, the median mucus layer thickness was significantly increased under flow (50 ± 24 vs. 29 ± 14 µm (static)), with a simultaneous accelerated maturation of the cells into a goblet-like phenotype. We demonstrated the strong impact of culture conditions on the differentiation and reorganization of HT29-MTX cells. The results comprise valuable advances towards the improvement of existing GI and mucus models or the development of novel systems using our newly designed culture chambers.

## 1. Introduction

The luminal surface of the gastrointestinal (GI) tract is a highly dynamic barrier consisting of a constantly renewing epithelial layer covered by mucus [1]. For a long time, the mucus was solely attributed to the protection against shear-induced damage and the invasion of pathogens. In recent years, this view has changed, and it was identified to be in a symbiotic relationship with the commensal microbiome; for example, by supporting the growth of indigenous bacteria, which, in exchange, deliver nutrients to the host [2]. For in vitro models, focusing on these host–microbiome interactions, a physiological mucus layer is fundamental [3,4]. Additionally, changes in the structure and composition of mucus are related to serious illnesses such as inflammatory bowel disease, although it is not yet clear whether they are the cause or consequence [5]. Therefore, an improved understanding of the dynamic mucus architecture is needed.

In general, mucus is a complex biological hydrogel, consisting mostly of water (~95%), proteins, salts, nucleic acids and cell debris. Depending on the location within the human GI tract, the thickness of the adherent mucus layer varies between 16 ± 5 µm and 155 ± 54 µm in the duodenum and rectum, respectively [6]. Its properties and structure change according to variations in pH-value, the concentration of calcium ions, and the action of DNases and pancreatic enzymes [7,8,9]. The structuring components are mucins, a family of highly O-glycosylated proteins, of which 21 members are known up to now. Mucins are divided into two groups: membrane-bound and secreted mucins. The latter are mainly represented by gel-forming mucins, which are able to form complex networks via multimerization through covalent and non-covalent interactions. The main gel-forming mucin in the stomach is MUC5AC, whereas MUC2 is predominant in the intestine [1]. To study the underestimated influence of mucus in health and disease, there is an urgent need for improved, reproducible cell-based mucus models.

Various mucus models have been published in the past, including ex vivo human and in/ex vivo animal models as well as diverging in vitro models. Even though the in vivo and ex vivo models are physiologically highly relevant due to their high degree of complexity, they suffer from intra- and inter-species variations, limited availability and ethical concerns [10]. These restrictions also apply to the use of raw mucus, for which not only batch-to-batch variations but also the involved isolation process cause high heterogeneity. Additionally, trapped enzymes in the isolated mucus can cause a degradation and aging over time [11]. Although the homogeneity of raw mucus can be increased by purification, it is time intensive and results in irreversible alterations of its native structure, composition and physical properties [12]. Alternatively, defined mucus biosimilars are under development, which is yet complicated by the high complexity of the natural example [13]. Instead of using the intricate in vivo models or raw mucus samples, the use of models based on mucus-producing cells circumvents most of the aforementioned limitations.

Notwithstanding the extensive developments in the culture of primary intestinal cells as organoids or monolayers, intestinal models based on immortalized cells currently remain the most commonly used systems for routine applications due to their robustness, scalability, reproducibility and cost-effectiveness, with Caco-2 cells being the most widely used cell line [14,15]. These cells are derived from a human colorectal adenocarcinoma and exhibit several properties of enterocytes, including the formation of tight junctions and the expression of drug transporters as well as typical metabolic enzymes [16]. However, their use for permeability studies suffers from several unphysiological factors, including an overly strong barrier formation with high transepithelial electrical resistance (TEER) values [17] and a lack of mucus production [18,19], which is accomplished in vivo by highly specialized goblet cells. Therefore, Caco-2 cells are commonly co-cultured with the human cell line HT29, also originating from a colorectal adenocarcinoma and differentiating into a constitutive mucus-forming phenotype in the presence of methotrexate (MTX) [20]. These co-cultures are extensively applied in in vitro models of the intestinal barrier [15,21], mimicking the physiological features somewhat more closely than monocultures. Nevertheless, studies focusing on the characterization of the resulting mucus layer, which is known to play an essential role in the reliability of these models [22,23], are scarce. When closer analysis was conducted, unphysiologically low mucus thicknesses, mostly in the range of a few micrometers, were found [17,24]. Accordingly, an increase in mucus production, and thus mucus thickness, could further improve the relevance of these models. Previously, the presence of biologically active molecules, such as prostaglandin E2, ATP or vasoactive intestinal peptide, as well as bacteria have been detected to induce elevated mucus generation and secretion [24,25,26]. Aside from that, the mucus formation can be increased by an underlying substrate or by culture under semi-wet or air-liquid-interface conditions [24,27]. Furthermore, several groups have reported the shear-induced mucus production of primary and Caco-2 cells in organ-on-a-chip systems [26,28,29,30]. These microfluidic devices are highly sophisticated, allowing, for example, other mechanical stresses in addition to the application of fluid flow, and were recently reviewed [31,32]. Despite all of the aforementioned benefits, these culture variants are still limited either by low mucus thicknesses, unphysiological culture conditions, costly supplementation or the requirement for complex equipment.

To overcome these restraints, we aimed to develop a simple millifluidic setup for the culture of cells under laminar flow conditions to thereby improve the mucus layer in a cell-based model without the need for further stimulation, making it easily accessible for deeper characterization of the mucus thickness and composition. Therefore, we developed a complementary set of reusable culture chambers that allow the application of physiological shear stress to cells on microscope slides or on membranes of commercially available cell culture inserts. Designed for this set of conventional solid and porous cell culture substrates, our chambers are intended to augment and refine state of the art microfluidic devices by providing the opportunity to apply homogenous shear stress in varying magnitudes on the entire macroscopic cellular seeding area. This versatility makes it possible to address a multitude of diverse scientific questions. We herein solely focused on the mucus-producing HT29-MTX cell line, hypothesizing that the applied mechanical influence is sufficient to promote their maturation into goblet-like cells and consequently increase mucus production. The obtained results could further improve its significance as a simple and readily available human mucus model or as component in future cell line-based GI models with higher physiological relevance. Thus, HT29-MTX cells were cultured under defined dynamic conditions and analyzed in terms of proliferation, differentiation, 3D reorganization, mucus generation and barrier formation. The composition of the adherent mucus layer was further characterized via immunofluorescent staining and its thickness was determined by confocal microscopy.

## 2. Materials and Methods

### 2.1. Materials

Dulbecco’s Modified Eagle’s Medium (DMEM, high glucose, GlutaMAX™ supplemented), trypsin-ethylenediamine tetraacetic acid (Trypsin-EDTA), Dulbecco’s phosphate-buffered saline (PBS), proliferation assay (Click-iT™ EdU Cell Proliferation Kit, Alexa Fluor™ 488), DNA-intercalating dye (Hoechst 33342), proteinase K powder (from Engyodontium album), 96 well plates (Black/Clear Bottom), amine-modified fluorescent latex beads (FluoSpheres™, 0.2 µm, yellow-green), MUC5AC-antibody (mouse, monoclonal), radioimmunoprecipitation assay buffer (RIPA, Lysis and Extraction Buffer), protease inhibitor (Halt™, EDTA-free) and ZO-1-antibody (rabbit, polyclonal) were purchased from Thermo Fisher Scientific (Waltham, MA, USA). Fetal bovine serum (FBS, Superior), non-essential amino acids (MEM-NEAA), octoxynol 9 (Triton™ X-100), alcian blue solution (1% in 3% acetic acid, pH 2.5), periodic acid Schiff (PAS) staining kit, MUC5B-antibody (rabbit, polyclonal), *p*-nitrophenyl phosphate solution (pNPP, Alkaline Phosphatase Yellow Liquid Substrate) and phalloidin–Atto^®^ 647N were obtained from Sigma-Aldrich (St. Louis, MO, USA). Microscope slides (Superfrost^®^), 4% paraformaldehyde (PFA, ROTI^®^Histofix 4%), tris-(hydroxymethyl)-amino methane (Tris, ≥99.3%, PUFFERAN^®^), EDTA (≥99), albumin fraction V (BSA, biotin-free) and sodium hydroxide (NaOH, ≥98%) were ordered from Carl Roth (Karlsruhe, Germany). Glacial acetic acid (>99.7) and hydrochloric acid (HCl, 37%) were purchased from Fisher Scientific (Loughborough, UK), 6-well tissue culture plates and rectangular cell culture dishes (quadriPERM^®^) from Sarstedt (Nümbrecht, Germany) and syringe filters (Minisart^®^, PTFE, 0.2 µm) from Sartorius (Göttingen, Germany). MUC1-antibody (rabbit, polyclonal) was obtained from Abcam (Cambridge, UK), polycarbonate (Makroclear^®^) from Arla Plast (Borensberg, Sweden), mounting medium (ProTaqs^®^ Mount Flour) from Biocyc (Luckenwalde, Germany), DNA-quantitation kit (AccuBlue^®^ Broad Range) from Biotium (Hayward, CA, USA) and antibiotic-antimycotic solution from Biowest (Nuaillé, France). Silicone tubings (Puri-Flex L/S 14^®^) were purchased from Cole Parmer (Vernon Hills, IL, USA), 12-well cell culture inserts (Transwell^®^, PET, 0.4 µm) from Corning (Corning, NY, USA), HT29-MTX-E12 from ECACC (Porton Down, Wiltshire, UK), thumbscrews (M3 × 10, DIN 646, stainless steel) from ERIKS Deutschland (Halle, Germany), 3D-printable resin (Dental SG Resin) from Formlabs (Somerville, MA, USA), round coverslips (Ø 18 mm No.1) from Glaswarenfabrik Karl Hecht (Sondheim v.d. Rhön, Germany), 35 mm dishes (high, glass bottom) from ibidi (Martinsried, Germany). Luer lock to M5 thread adapters (stainless steel) were obtained from Key Surgical (Lensahn, Germany), Luer lock to barb connectors (polypropylene) from QOSINA (Ronkonkoma, NY, USA), MUC2-antibody (mouse, monoclonal) from Santa Cruz Biotechnology (Dallas, TX, USA), duplicating silicone (REPLISIL 22 N) from SILCONIC^®^ (Lonsee, Germany), 3-[(3-Cholamidopropyl)dimethylammonio]-1-propansulfonat (CHAPS, VWR International, Darmstadt, Germany) and heat-seal sterilization bags (400 mm × 250 mm) from SÜDPACK Medica (Baar, Switzerland).

### 2.2. Chamber Design and Fabrication

#### 2.2.1. Slide Chamber for Dynamic Culture on Solid Substrates

**Figure 1:**
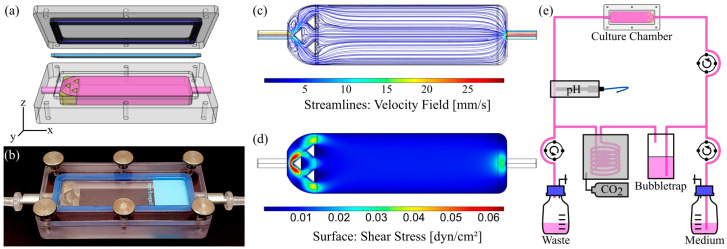
Chamber design, evaluation of flow properties and bioreactor setup for dynamic culture of cells on solid substrates. (**a**) CAD model (exploded view) and (**b**) photograph of the assembled culture chamber with 6 mm channel height and flow homogenizing insert. (**c**) Corresponding velocity streamline profile within the fluid channel and (**d**) surface shear stress obtained by CFD simulation at a volume flow rate of 6.4 mL∙min^−1^. (**e**) Schematic illustration of the bioreactor circuit.

The culture chambers were computer-aided designed (CAD) with Rhinoceros (Version 5.0, Robert McNeel & Associates, Seattle, WA, USA). The chambers consist of a body, which fits and holds a standard 26 mm × 76 mm × 1 mm microscope slide in its cavity and forms a flow channel of defined height when closed with a lid (Figure 1a). Fluid tight sealing of the chambers was accomplished by the insertion of casted gaskets made from medical grade silicone in between the body and the lid, and the closing of the chambers with six knurled thumb screws. The applicable wall shear stress *τ* was estimated using the following Equation (1):

*τ* = 6*µQw*^−1^*h*^−2^, (1)

with the dynamic viscosity *µ*, the volume flow rate *Q* as well as the width *w* and height *h* of the fluidic channel, which was varied from 0.15 to 6 mm (Table S1). The chambers were fabricated from bulk polycarbonate by conventional CNC-milling. A homogenizing triangular structure was 3D-printed from a commercially available, biocompatible and autoclavable ink for the chamber with a 6 mm channel height (see Supplementary Materials for detailed information on 3D-printing, CFD-simulation and material biocompatibility assessment). This chamber, with a homogenizing structure implemented, was used for dynamic culture on solid substrates. After fabrication, threads were cut and steel adapters were mounted to connect hoses. All materials remained inherently stable after autoclaving for at least 10 cycles.

#### 2.2.2. Insert Chamber for Dynamic Culture on Membranes

The insert chamber was completely CAD-modeled and subsequently printed with the aforementioned resin. The chamber fits a 12-well Transwell^®^ cell culture insert and exhibits a flow channel with 3 mm height. The channel was designed to rise from the inlet to the outlet, allowing potential air bubbles in the circuit to be removed by the fluid flow. Silicone O-rings were used to seal the lid and Luer adapters were used to connect the fluid in- and outlet. An illustration and the realization of the chamber are shown in Figure 7a,b.

#### 2.2.3. Computational Fluid Dynamics (CFD)

The numerical analysis of flow conditions within the designed culture chambers was carried out with COMSOL Multiphysics (v5.5, including the CFD-module, COMSOL, Stockholm, Sweden). The inlet flow properties were set to 6.4 mL∙min^−1^ and 1.42 mL∙min^−1^ for the slide and insert chamber, respectively. Zero pressure was defined as outlet condition. Gravity was included and the dynamic viscosity *µ* of the fluid was set to 0.93 cP [33]. Meshing was performed using the “physics-controlled”-mesh option as free tetrahedral elements and the mesh-quality was judged based on COMSOL’s minimum element quality.

### 2.3. Cell Culture

Human intestinal HT29-MTX cells (ECACC, Porton Down, UK) were cultured in DMEM High Glucose medium, supplemented with 10% FBS, 1× MEM-NEAA and 1× antibiotic-antimycotic solution at 37 °C and 5% CO_2_ in a humidified atmosphere. Trypsin-EDTA (0.05%) was used to passage cells once a confluency of 60–80% was reached. The seeding density for all experiments was 2 × 10^5^ cells∙cm^−2^. The passage number of cells used in experiments ranged from 24 up to 36.

#### 2.3.1. Solid Substrates

For static experiments, cells were seeded on 6-well plates, on 35 mm dishes with glass coverslip bottom for microscopy or on glass coverslips for the determination of thickness. Medium exchange was performed every two to three days. Prior to dynamic culture, cells were seeded on sterilized glass coverslips for the determination of thickness, or on glass microscope slides in rectangular cell culture dishes with the aid of silicone casted cell separators to define a growth area of 2 × 3 cm^2^ per slide. Separators were removed 24 h after cell seeding and cells were cultured for two more days under static conditions to reach confluency.

#### 2.3.2. Membranes

Cells were seeded either conventionally into the Transwell^®^ inserts on the top of the membranes, or on the bottom of the membrane, which will be referred to as “normal” and “reverse” culture below. For reverse culture, cell culture inserts were flipped, equipped with sterile cast silicone rings and transferred into 6-well plates. After overnight adhesion, the inserts were transferred into the corresponding 12-well plate. The medium volume was 0.5 mL in the upper and 1.5 mL in the lower compartment.

### 2.4. Dynamic Cell Culture

#### 2.4.1. Bioreactor Setup for Dynamic Culture on Solid Substrates

The stand-alone bioreactor system (OSPIN, Berlin, Germany) for dynamic culture comprises peristaltic pumps for medium exchange and perfusion, a pH sensor to monitor and adjust the pH-value, and temperature control via the circulation of heated air. The mounted peristaltic pumps enable a constant perfusion rate of 2.84 to 14.2 mL∙min^−1^ when operated at 20 to 100 RPM with silicone hoses with an inner diameter of 1.6 mm. A schematic illustration as well as a photograph can be found in Figure 1e and Figure S3a, respectively.

For sterilization of all parts, the assembled culture circuit was transferred into sterilization pouches, which were sealed and autoclaved for 20 min at 121 °C and 2 bar. Afterwards, the reservoir flask of the assembled circuit was filled with fresh medium under sterile conditions and sealed with sterile filters for gas exchange. The circuit was integrated into the bioreactor and equilibrated for 24 h at a constant fluid flow rate of 6.4 mL∙min^−1^ (45 RPM). Subsequently, confluent cells on microscope slides or glass coverslips were transferred into the chamber with 6 mm channel height and cultured under the aforementioned conditions. A complete medium exchange of approximately 40 mL was performed every four to five days. The applied flow rate generates a physiological surface shear stress of 0.009 dyn∙cm^−2^ (9 × 10^−4^ Pa) on the cellular monolayer. The described conditions are referred to as “dynamic culture conditions”.

#### 2.4.2. Bioreactor Setup for Dynamic Culture on Membranes

The dynamic culture of cells on inserts was carried out using a peristaltic pump (Model 114 ST, Watson Marlow, Falmouth, UK) in a cell culture incubator under standard conditions (37 °C, 5% CO_2_). The mounted stepper motor was operated with an Arduino UNO R3 microcontroller with the use of the AccelStepper library v1.61. The circuit consisted of four series-connected chambers and a bubble trap, shown in Figure S3b. Sterilization was performed as described above. The pump was operated at 10 RPM, generating a constant fluid flow of 1.42 mL∙min^−1^, which results in an average surface shear stress of 0.012 dyn∙cm^−2^ (12 × 10^−4^ Pa). The entire culture medium of 12 mL was exchanged every four days.

### 2.5. Cell Proliferation Assay

The proliferation of cells was detected via 5-Ethynyl-2′-deoxyuridine (EdU) incorporation according to the manufacturer’s instructions. Briefly, cells were incubated with 10 µM EdU in cell culture medium for 2 h under static or dynamic conditions. Cells were fixed with 4% PFA for 10 min, permeabilized with 0.5% Triton-X 100 for 20 min and afterwards incubated with the EdU-Click-ItTM reaction cocktail for 20 min. Nuclei were counterstained with 1 µg∙mL^−1^ Hoechst 33342 in PBS for 15 min in the dark. Finally, cells were mounted and analyzed via confocal microscopy (LSM800, Carl Zeiss, Jena, Germany) or via epifluorescence microscopy (Axio Observer Z1, Carl Zeiss) equipped with a black and white camera (AxioCam MR R3, Carl Zeiss). All steps were conducted at room temperature.

### 2.6. Mucus Staining and Quantification

To visualize acidic mucins, samples from dynamic and static culture were fixed using PFA, washed with PBS and incubated with Alcian Blue G8x Solution (1% in 3% acetic acid) for 15 min at room temperature. Subsequently, the solution was removed and samples were washed with distilled water, followed by washing steps with 3% acetic acid and water to remove residual staining solution. To stain neutral mucins, fixed samples were incubated for 2 min with 0.5% periodic acid, washed and treated for 10 min with Schiff’s reagent, followed by three washing steps with PBS. Stained samples in PBS were imaged using an inverted microscope (Axio Observer Z1, Carl Zeiss) equipped with a color camera (AxioCam 105 color, Carl Zeiss).

To quantify neutral mucins, cells and adherent mucus were removed from the culture substrate using a cell scraper, vigorously resuspended in PBS (1 mL), snap frozen in liquid nitrogen and stored at 80 °C until further analysis. The cell suspension (100 µL) and 2% *w*/*v* CHAPS (100 µL) were mixed and diluted with PBS (800 µL). The resulting suspension was sequentially incubated with periodic acid (20 µL) and Schiff’s reagent (100 µL) for 2 and 1 h, respectively, at 37 °C in a thermal shaker. The absorbance A_555_ of the resulting solution (100 µL) was measured in 96-well plates using a microplate reader (Infinite™ M200 PRO, Tecan, Männedorf, Switzerland).

For normalization, DNA content was determined using AccuBlue^®^ assay according to the manufacturer’s instructions. In brief, the cell suspension (100 µL) was resuspended in 2× digestion buffer (20 mM Tris, 2 mM EDTA, 0.2% Triton X-100 and 1 mg∙mL^−1^ proteinase K) and incubated overnight at 37 °C. AccuBlue^®^ reagent was diluted 1:100 in reaction buffer, mixed with the sample at a ratio of 10:1 and incubated for 15 min in the dark. Samples were excited at 480 nm and emission E_520_ was measured in 96-well plates using a microplate plate reader in reference to a standard calibration curve.

### 2.7. Thickness-Determination of the Adherent Mucus Layer

The thickness of the adherent mucus layer was determined as previously described in the literature using fluorescent particles and confocal microscopy [34,35]. In brief, HT29-MTX cells, on coverslips, were incubated with amine-modified, fluorescent latex beads (Ø 200 nm, 1:500) and Hoechst 33342 (5 µg∙mL^−1^) in medium for 20 min under cell culture conditions. Samples were gently washed twice with warm PBS and afterwards analyzed in cell culture medium via confocal microscopy. The ImageJ based software package Fiji v1.52 [26] was used for image processing and analysis to estimate the median mucus thickness (± median absolute deviation, MAD, *n* = 5) of the adherent hydrated mucus layer. Detailed information about image processing and data acquisition can be found in the Supplementary Materials (Figure S4).

### 2.8. Alkaline Phosphatase (ALP) Activity Assay

To determine intracellular ALP activity, HT29-MTX cells were removed from their substrate using a cell scraper, washed with PBS and centrifuged for 10 min at 5000× *g*. PBS was aspirated, cells were resuspended in cold RIPA buffer (60 µL), containing 1× EDTA-free protease inhibitor and incubated for 40 min on ice. Reaction tubes were briefly vortexed every 10 min to ensure complete cell lysis. After incubation, the lysates were centrifuged for 5 min at maximum speed to sediment insoluble cell debris. The supernatant and ALP working reagent were mixed at a 1:1 ratio in a 96-well plate. The ALP-catalyzed formation of *p*-nitrophenol was colorimetrically monitored over a period of 60 min by measuring the change of absorbance as Δ(A_405_−A_660_)∙min^−1^ with a microplate reader.

### 2.9. Immunostaining and Direct Labelling

Cells were fixed with 4% PFA for 20 min, permeabilized with 0.1% Triton X-100 for 10 min, blocked using 5% BSA in PBS for 90 min and afterwards incubated with primary antibodies against MUC1, MUC2, MUC5AC, MUC5B or ZO-1 for 4 h. Primary antibodies were detected via incubation with fluorophore-coupled secondary antibodies for 90 min (see Table S3 for further information). All samples were counterstained with Hoechst 33342 (10 ng∙mL^−1^) in PBS, and selected samples were counterstained using phalloidin-Atto^®^647, for 15 min in the dark. All steps were performed at room temperature, samples were analyzed via confocal microscopy and images were processed with the Zen Blue software (v2.3).

### 2.10. Transepithelial Electrical Resistance (TEER)

The electrical resistance of HT29-MTX cells seeded on Transwell^®^ inserts was measured using the Millicell^®^ ERS-2 unit equipped with an STX1 electrode (Millipore, Bedford, MA, USA) to assess their barrier function. To ensure accurate measurements, apical and basolateral medium was replaced by pre-warmed, fresh medium and inserts were kept on a 37 °C heating plate (HP062, AgnThos, Lidingö, Sweden) for 10 min prior to and during the measurement. Cell culture inserts without cells were measured as reference.

### 2.11. Statistics

All experiments were conducted at least in triplicate with a minimum of two technical replicates. Statistical significance between two groups was evaluated using the Wilcoxon/Mann–Whitney test, whereas statistical significance between more than two groups was evaluated using the Kruskal–Wallis test, followed by Dunn’s post-hoc test with OriginPro 2020b (v9.7.5.184, OriginLab, Northampton, MA, USA). Significances are indicated as * *p* ≤ 0.05; ** *p* ≤ 0.005 and *** *p* ≤ 0.0005.

## 3. Results and Discussion

### 3.1. Characterization of Flow Chamber for Solid Substrates

To culture cells on standard microscope slides under defined dynamic conditions, an easy to reproduce, reusable flow chamber was designed and manufactured (Figure 1a,b). It allows implementation into a stand-alone bioreactor system, as illustrated in Figure 1e and Figure S3a. All materials remained inherently stable after repeated cycles of autoclaving. Additionally, the chamber was operable in a common CO_2_ incubator with a peristaltic pump that was similar to the setup of the flow chambers for culture of cells on membranes shown below (Figure S3b).

The geometry of the chamber was shaped to generate homogenous laminar flow on the cell surface with adjustable physiological shear stresses. By varying the height of the flow channel from 0.15 to 6 mm, we mathematically approximated an applicable range of wall shear stresses from 0.004 to 29.7 dyn∙cm^−2^ (4∙10^−4^ to 2.97 Pa; Equation (1)), which resembles the physiological shear stresses of a variety of human organs and blood vessels [36], opening up the opportunity to cultivate cells from various origins under their respective physiological flow conditions to generate more realistic tissue mimicking models [37]. The obtained values were further validated and the fluid flow behavior within the chambers was characterized via CFD-simulations, which revealed differences between simulated and calculated values, with the latter being slightly overestimated (Table S1). Chambers with channel heights of up to 1.5 mm already exhibited laminar flow conditions due to a construction-dependent threshold (Figures S1a and S2). As it can be seen from Figure S1b, homogenous laminar flow necessitated the implementation of homogenizing structures for chambers with 6 mm channel height, which were designed on the basis of the conducted CFD simulations (Figure S1c). A representative example, including the velocity profile and surface shear stress, is shown in Figure 1c,d for a corresponding chamber with 6 mm channel height and 3D-printed homogenizing triangular features at a volume flow rate of 6.4 mL∙min^−1^. The estimated and simulated surface shear stresses were 0.008 and 0.009 dyn∙cm^−2^ (8 and 9 × 10^−4^ Pa), thus matching with physiological shear stresses in the human small intestine [28]. Similar shear stresses could be applied using convenient ibidi^®^ chambers (µ-Slide I, 0.8 mm channel height), which in preliminary experiments unfortunately resulted in a marked pH-gradient from the inlet to the outlet with the HT29-MTX cells under physiological flow conditions (data not shown). Overcoming these limitations, our newly designed flow chamber was the better choice for the dynamic culture of HT29-MTX cells when aiming to develop a more realistic in vitro mucus model of the human GI tract.

### 3.2. Design of Experiments

The human cell line HT29-MTX generally requires 21 days to fully differentiate into an intestinal epithelial, mucus-producing cell layer under static conditions [38]. Their ongoing maturation into functional goblet-like cells is macroscopically visible, since the mucus layer, accumulated on top of the cell layer, progressively reduces its transparency. It was previously shown that the mucus production of HT29-MTX cells cultured on membranes at the liquid–air interface is enhanced under mechanical stimulation [24,39]. Preliminary experiments revealed a markedly faster and stronger development of the aforementioned visual turbidity of the cultures under flow conditions using our chamber, which suggests a reduced culture time of only 14 days under dynamic conditions to yield a mucus-producing epithelial cell layer (data not shown). Hence, the maturation of HT29-MTX cells in terms of proliferation, differentiation and mucus production under static and dynamic conditions was further characterized at different time points up to three weeks of culture.

### 3.3. Morphology and Proliferation of HT29-MTX Cells under Static and Dynamic Conditions

**Figure 2:**
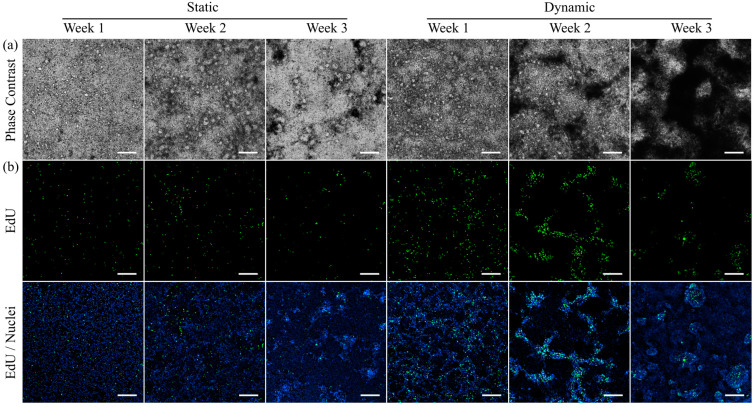
Morphology and proliferation of HT29-MTX cells under static and dynamic culture conditions at various time points. (**a**) Morphology was assessed via phase contrast microscopy and (**b**) newly synthesized DNA was visualized with EdU-Alexa^®^ 488 (green) and imaged using confocal microscopy. Nuclei were counterstained with Hoechst 33342 (blue). (**a**,**b**) show independent samples. (Scale bars: 200 µm).

To monitor differences in the development of integrity and morphology of HT29-MTX cells under static and dynamic culture conditions, the conformation of the cell layer was first monitored at different time points via phase contrast microscopy (Figure 2a). The cell layers were confluent at all times and conditions without any recognizable defects. Additionally, we observed a time-dependent formation of darker regions that are hard to bring into focus during microscopic imaging. This phenomenon developed faster and was distinctly more pronounced under dynamic conditions, which was in agreement with the macroscopic observation of turbidity when compared to samples from static conditions at the same or even advanced time points. It was previously reported in the literature that HT29-MTX monolayers are able to expand three-dimensionally (3D) [27]. The presence of multilayered regions and the accumulation of mucus seem to decrease the light transmission in the respective areas and hinder optimal focus adjustment.

To further examine this effect, the influence of culture conditions on the proliferative capacity of HT29-MTX cells was detected via incorporation of the thymidine analogue EdU into the newly synthesized DNA of daughter cells, and subsequent labelling (Figure 2b). Regardless of their cancerous origin, the proliferative capacity of HT29-MTX cells is known to disappear once cells reach confluency [40]. However, a larger number of proliferating cells was detected at all analyzed time points under dynamic conditions compared to samples from static culture. These results are consistent with the observed increased proliferation of Caco-2 cells, when cultured under flow conditions [41]. Furthermore, the simultaneous visualization of nuclei via Hoechst-staining revealed HT29-MTX clusters with a higher fluorescence intensity, which were particularly defined after two and three weeks of dynamic culture when compared to the status after three weeks of static culture. The clusters share structural similarities with the dark spots on the cell layer detected via phase contrast microscopy and can be attributed to multiple layers of highly proliferative cells and their 3D rearrangement beyond cellular monolayers. Proof of the colocalization of the darker regions and proliferative cells is shown in Figure S5 using epifluorescence microscopy. To exclude a potential influence of the larger medium volume, and thus, constant and high nutrient supply present in dynamic culture, control experiments using equally high medium volumes in static culture were conducted, revealing no effect on the proliferation of the HT29-MTX cells (Figure S7). The improved proliferation and 3D organization under dynamic culture conditions seem to be solely induced by the applied physiological shear stress.

### 3.4. Villi Formation

**Figure 3:**
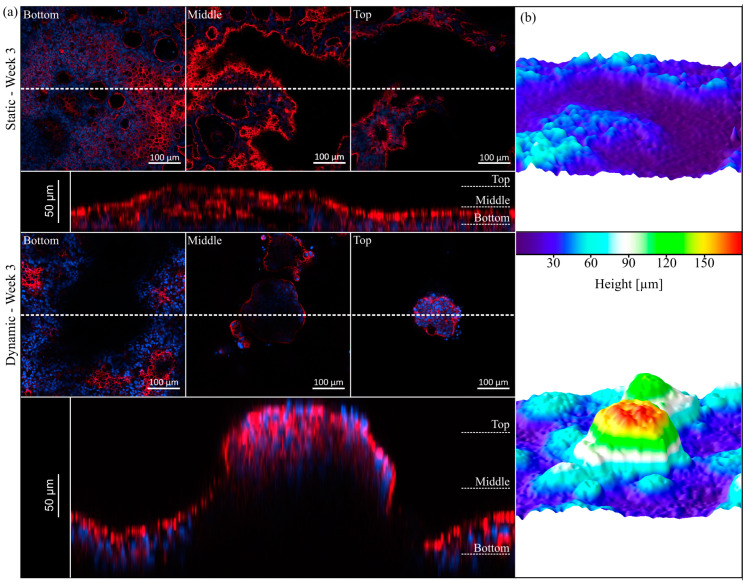
Topography of HT29-MTX cells under static and dynamic conditions after three weeks of culture. (**a**) Confocal images of F-actin (red) and nuclei (blue) staining at different z-positions (Bottom, Middle, Top) and representative orthogonal views below (the dotted line indicates the cutting plane). (**b**) Corresponding 3D surface reconstructions of z-stacks of the epithelium, modelled by ImageJ.

The presence of intestinal micro- and macrovilli and the accompanying increase in overall surface area determines the high absorptive capacity of the small intestine [42]. Based on the previous data on morphology and proliferation, we further examined the cellular reorganization via visualization of the cytoskeleton. The 3D expansion of HT29-MTX cells under static and dynamic conditions can be seen in Figure 3a. For both culture conditions, hollow structures are visible in the lower regions of the epithelial layer, which are covered by cells on the top. In the literature, such structures within the HT29-MTX cell layer were previously described as arch morphology or mucus-containing vacuoles without attribution of a function [24,27]. In our hands, these structures did not contain mucins, but increased over time in number and size, merged to form larger entities and were overgrown by cell layers, resulting in a 3D expansion of the cellular monolayer. Although not yet completed, this 3D reorganization was more pronounced after two weeks of dynamic culture, with the villi-like structures having a height of up to 120 µm [43], when compared to the situation after three weeks of static culture, in which there was a height of only up to 80 µm. After three weeks of dynamic culture, the villi-like structures were even more defined and completely singularized, reaching up to 180 µm in height (Figure 3a). These results validate the faster and much more sufficient maturation and reorganization of the cellular layer under minimal flow conditions.

### 3.5. Mucus Characterization

**Figure 4:**
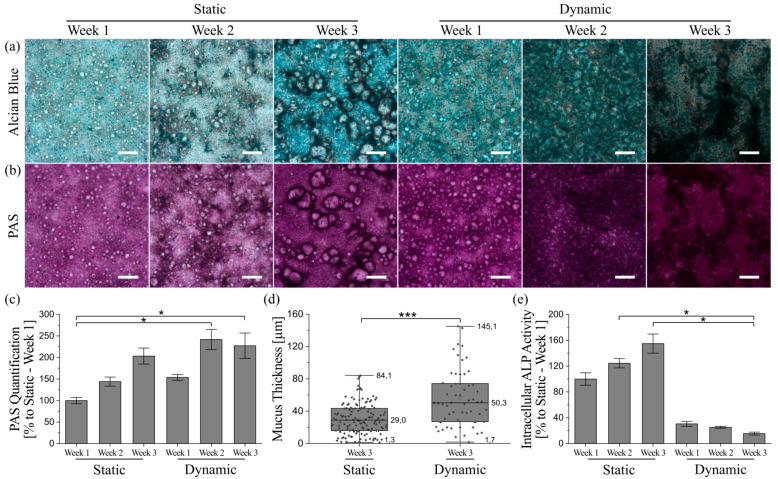
Qualitative and quantitative characterization of mucus produced by HT29-MTX cells under static and dynamic culture conditions at various time points. Microscopy images of (**a**) Alcian blue and (**b**) PAS-stained cells showing acidic mucins in blue and neutral mucins in purple, respectively. (Scale bars: 200 µm). (**c**) Photometrically quantified amounts of neutral mucins after PAS-incubation. Data presented as mean ± SEM with respect to week 1 under static conditions (*n* = 3–5). (**d**) Adherent mucus layer thickness determined under native conditions via confocal imaging data, shown as box plot, including data points, median and full range (*n* = 5). (**e**) Intracellular activity of ALP as enterocyte marker. Data presented as mean ± SEM with respect to week 1 under static conditions (*n* = 3–8). Statistical significance indicated by * *p* ≤ 0.05 and *** *p* ≤ 0.0005.

Mucus synthesis and secretion, a major function of the intestinal epithelium [1], was examined for both culture conditions. Staining of acidic and neutral mucins revealed a time-dependent and ongoing increase in the respective mucins under static and dynamic culture conditions (Figure 4a,b). The staining intensified more rapidly under dynamic conditions, which was not due to the higher medium volume and, therefore, better nutrition of the cells in this culture system. According to Wang et al., an increased mucus formation and thickness could be the result of a reduced dilution of mucin molecules in a smaller medium volume [25]. In our hands, the higher dilution in dynamic culture nevertheless resulted in increased mucus formation, whereas the same medium amount applied to static culture conditions revealed a dilution of mucins compared to the conventional static conditions (see Figure S8a). The higher amount of mucus per cell in dynamic culture can, therefore, be solely related to the applied fluid flow. To achieve the thickest possible mucus layers under static conditions, we recommend a reduction in medium volume (by as much as practically feasible) and particularly careful media changes.

Furthermore, both acidic and neutral mucins seem to be located preferentially close to the aforementioned villi-like structures, which becomes most obvious under static conditions. Because the presence of multicellular layers is also accompanied by a reduction in visible light transparency, the color images of the stained samples might misleadingly suggest higher amounts of mucins at cell dense positions with 3D villi-like structures. Thus, we additionally determined the amount of neutral mucins per cell and were able to confirm the time-dependent increase in mucus production under both culture conditions (Figure 4c). The only significant increases in mucin amount were detectable after two and three weeks of dynamic culture when compared to static conditions after week 1. The 2.4-fold increase in mucin generation after two weeks of dynamic culture was already higher than the 2-fold increase after three weeks of static culture, clearly demonstrating the accelerated differentiation of HT29-MTX cells into goblet-like mucus-producing cells induced by shear stress. After week 2, the mucin amount in dynamic culture remained relatively stable. This indicates the completion of differentiation into a mucus-producing phenotype after two weeks of physiological fluid flow, followed by a more defined 3D-restructuring of the cellular layers, as shown previously.

Adherent mucus is commonly analyzed after fixation of the respective samples, which causes the lack of data on mucus characteristics under native conditions. Unfortunately, common fixation methods lead to dehydration, and thus, a collapse of the adherent mucus, which does not represent its native structure. Consequently, the conventional fixation decreases the mucus thickness, resulting in thickness values in the range of a few micrometers for fixed HT29-MTX cells [17,24,27]. Even though there are some fixatives used that supposedly preserve its native structure, this is mutually dependent on the sample origin as the adherent mucus may be altered by multiple washing steps and further processing [44]. To overcome this obstacle, we incubated the native cultures with amine-modified fluorescent beads, which stick to the charged mucus via electrostatic interactions, and counterstained the cell nuclei with Hoechst. Confocal imaging and further image processing of z-stacks made it possible to measure the average distance between nuclei and beads sticking to the mucus surface (Figure 4d). We observed native mucus thicknesses reaching up to 84 µm after three weeks under static conditions, and 145 µm after three weeks under dynamic conditions. Thus, culture under physiological shear stress yielded an increase in maximum mucus thickness by 72%. The corresponding averaged thicknesses after three weeks of static or dynamic culture were 29 ± 14 µm and 50 ± 24 µm, respectively, indicating a significant average mucus thickness increase of 72% for flow conditions. Along with the accelerated development of mucus amounts in dynamic compared to static culture discussed above, a maximal mucus thickness of 148 µm and an average thickness of 41 ± 14 µm measured after only two weeks of culture under flow [43] confirm the assumption of a faster differentiation of HT29-MTX cells into a goblet-like cell type under physiological shear stress.

Additionally, the intracellular activity of the enterocyte marker enzyme alkaline phosphatase (ALP) was measured to evaluate the differentiation status of the cells under different culture conditions in greater detail [45]. As illustrated in Figure 4e, the ALP activity increases time-dependently in samples obtained from static culture. Cells derived from dynamic culture, on the other hand, show a drastic reduction in ALP activity from week 1 onwards. Static control experiments with media volumes comparable to dynamic culture also revealed a decrease in intracellular ALP activity after three weeks when compared to conventional static culture (Figure S8b). However, the ALP activity in samples from dynamic culture was still reduced by a factor of two, supporting the hypothesis of a better maturation of the cells towards a goblet-like phenotype under flow.

### 3.6. Immunofluorescent Staining of Selected Mucins

**Figure 5:**
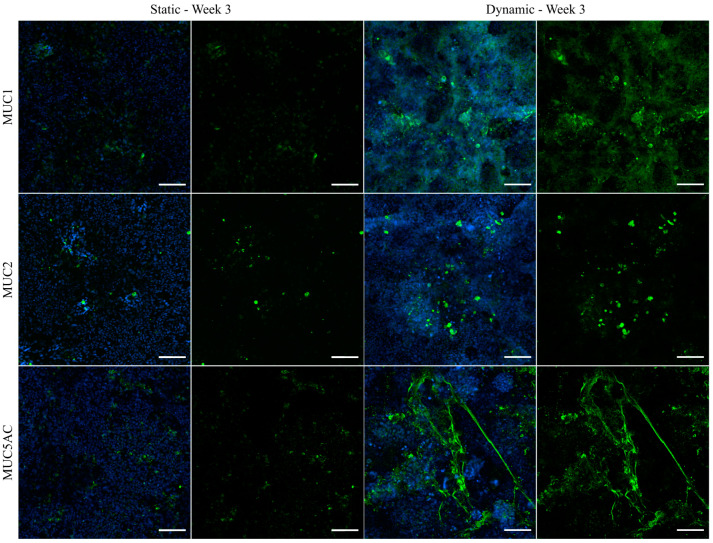
Confocal immunofluorescence images of HT29-MTX cells under static and dynamic conditions after three weeks. Samples were stained with antibodies for the membrane-bound mucin MUC1 and secreted mucins MUC2 and MUC5AC (green). Nuclei were counterstained with Hoechst 33342 (blue). (Scale bars: 100 µm).

To investigate the distribution and localization of selected mucins, the best characterized transmembrane mucin MUC1 and the two main GI secreted mucins MUC2 and MUC5AC were visualized via immunofluorescence (Figure 5). In general, all examined mucins were expressed at higher levels after culture of the cells under dynamic conditions and they were mainly located at the tips of the 3D villi-like structures, as can be concluded from the images showing the counterstaining of the nuclei. An enhanced shear-induced polarization was shown previously for various cell types, including epithelial and endothelial cells of different origins [24,46]. Furthermore, mucins are known to protect the underlying epithelium from mechanical stress [1], which is expected to be highest at the tip of the villi-like structures under dynamic conditions. MUC1 was hardly detectable under static conditions, whereas the tips of the villi-like structures of dynamically cultured cells were apically covered by MUC1. The stress-induced production of MUC2 was shown previously for HT29-MTX cells [27], and also for Caco-2 cells, which usually do not express MUC2 [47]. In our experiments, MUC2 seemed to be mainly stored intracellularly (Figure 5). MUC5AC is known to be highly expressed in the stomach and the respiratory tract [48] and it is the main secreted mucin of HT29-MTX cells [34]. It can multimerize by forming disulfide bonds and contributes to the formation of a dense mucus network [49]. As can be seen in Figure 5, under dynamic culture conditions, the secreted MUC5AC forms a heavily branched network, which seems to span the tips of the villi-structures and as such may contribute to an increased robustness of the formed mucus layer. The immunofluorescent staining allows the observation of an increased mucus coverage of the cellular layer after culture under flow conditions. Additionally, the increased generation of secreted mucins after culture of the cells for only two weeks under dynamic conditions, compared to a culture spanning three weeks under static conditions, confirms the accelerated maturation of the HT29-MTX cells in the direction of the goblet cell phenotype. The respective images of the staining from all of the various time points, including week 2 of dynamic culture, as well as a further immunofluorescent staining for MUC5B can be found in Figure S6. Again, a control experiment using the medium volume of dynamic culture in a static approach resulted in a dilution of mucins even when compared to static culture (Figure S9). All effects seen under dynamic culture conditions can, therefore, be attributed to the influence of the fluid flow applied to the cell surface.

**Figure 6:**
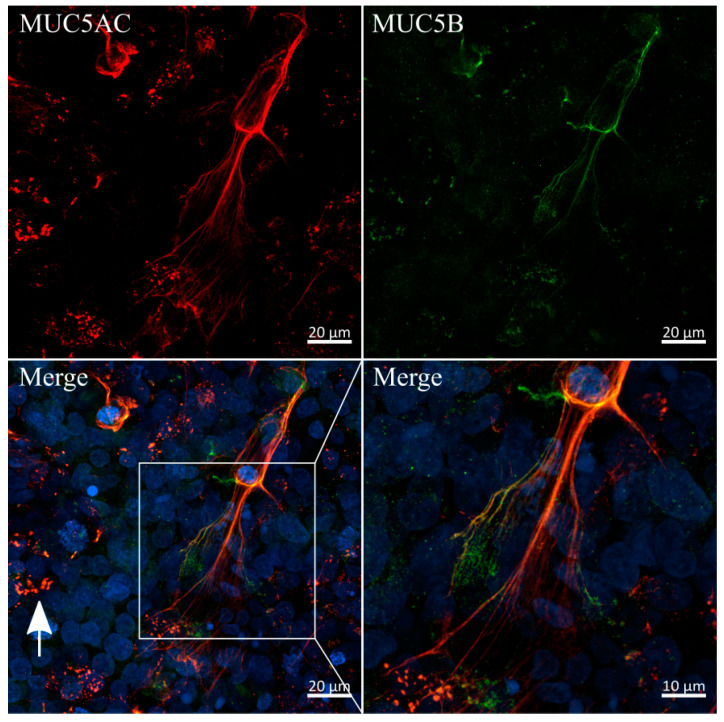
Colocalization and fiber-formation of secreted mucins MUC5AC and MUC5B. Confocal immunofluorescence images of HT29-MTX cells after two weeks of dynamic culture stained with antibodies for MUC5AC (red) and MUC5B (green). Nuclei were counterstained with Hoechst 33342 (blue). White arrow indicates an example of colocalization of both mucins in secretory vesicles.

Since the immunofluorescent staining of MUC5AC showed high similarities to mucus networks found on the surface of airway epithelia, previously reported by Sheehan et al., we conducted a co-staining of MUC5AC and the main secreted airway mucin 5B (Figure 6) [50]. Interestingly, both mucins were colocalized in the previously observed branched networks and, to a lesser extent, in secretory vesicles, as indicated by the white arrow. Imaging at higher magnifications revealed diameters for these single fibers as small as 200 nm. A similar colocalization of fibrous MUC5AC and MUC5B in mucus networks is commonly observed in lung-derived mucus samples [51]. To the best of our knowledge, our results are the first to show a fibrous and heavily entangled network of MUC5AC and MUC5B strands produced and secreted by the intestinal HT29-MTX cell line.

### 3.7. Transfer of the Model System from Solid Substrates to Membranes

**Figure 7:**
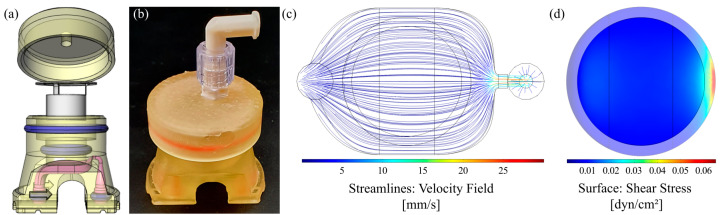
Chamber design for dynamic culture of cells on membranes and evaluation of flow properties. (**a**) CAD model (exploded view) and (**b**) photograph of the assembled 3D-printed culture chamber. (**c**) Corresponding velocity streamline profile within the fluid channel and (**d**) surface shear stress highlighted for the membrane area obtained by CFD simulation at a volume flow rate of 1.42 mL∙min^−1^.

In order to transfer the established well-defined dynamic cell culture conditions for the fast maturation and differentiation of HT29-MTX cells from the microscopic slides to commercial cell culture inserts, we developed a fully 3D-printed insert culture chamber that enables the culture of cells on membranes under flow conditions. A similar setup is commercially available, comprising a silicone-based chamber that allows the application of apical and basal shear stress. However, silicone is known to adsorb hydrophobic drugs and applicable shear stresses are restricted to a relatively low maximum flow rate (≤500 µL/min) with this system [52]. Furthermore, CFD simulations revealed that rather non-homogenous shear stresses were applied to the cellular surface, originating from the chamber design [53]. The CAD-model of our design and the printed and assembled chamber are shown in Figure 7a,b. CFD simulation at a volume flow rate of 1.42 mL∙min^−1^ (corresponding to 10 RPM) demonstrated a homogenous flow distribution, as illustrated by the velocity field, which applies a physiological shear stress of 0.012 dyn∙cm^−2^ (12 × 10^−4^ Pa) to the cell surface of the cell culture insert (Figure 7c,d). The Dental SG Resin (Formlabs) used for 3D-printing of the insert chamber is specified as biocompatible. Nevertheless, a leaching assay was performed, which revealed no influence on the viability and no increase in cell death of HT29-MTX cells induced by contact of the cell culture medium with the printed resin for up to three weeks (Figure S10).

**Figure 8:**
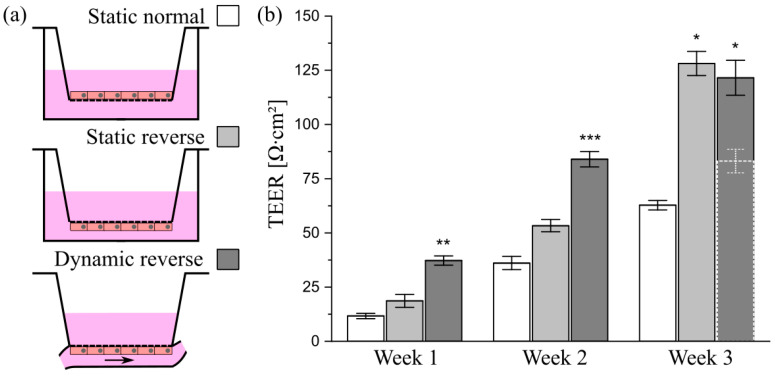
Influence of culture conditions on the barrier function of HT29-MTX cells. (**a**) Illustration of culture conditions with cells seeded either onto the top or bottom of Transwell^®^-insert membranes, termed as “normal” and “reverse”, and cultured under static or dynamic conditions. (**b**) Barrier function of HT29-MTX cells cultured under “Static normal” (white), “Static reverse” (light gray) and “Dynamic reverse” (dark gray) condition as quantified by TEER measurements at various time points. Data presented as mean ± SEM (*n* = 4–12). Dotted bar (Dynamic reverse—Week 3) represents raw data prior to correction of the value for the surface area. * *p* ≤ 0.05; ** *p* ≤ 0.005 and *** *p* ≤ 0.0005 compared to corresponding “Static normal”.

**Figure 9:**
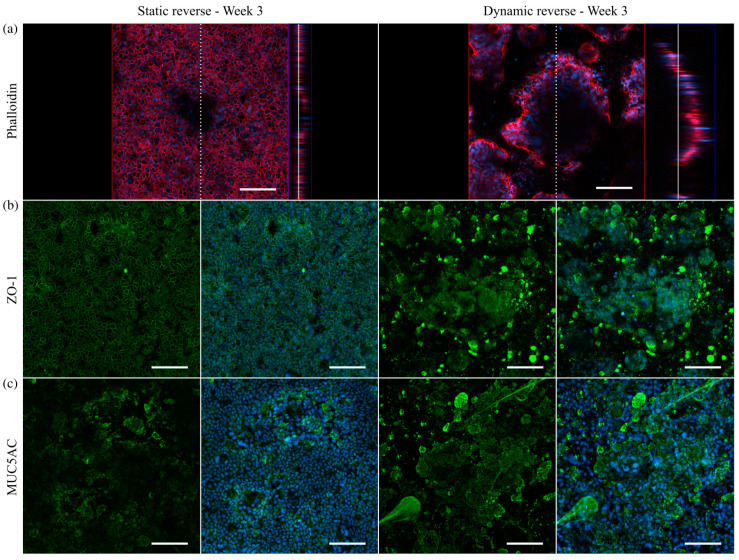
Confocal immunofluorescence images of HT29-MTX cells on Transwell^®^-insert membranes under static and dynamic conditions after three weeks. (**a**) Confocal image and corresponding orthogonal view of F-actin stained with phalloidin (red). Dotted and straight white line indicate the cutting plane and the position within the z-stack, respectively. Nuclei were counterstained with Hoechst 33342 (blue). Orthogonal projections of samples stained with antibodies for (**b**) ZO-1 as an integral part of tight junctions (green) and (**c**) the secreted mucin MUC5AC (green). (Scale bars: 100 µm).

To ensure that cells cultured on conventional inserts are accessible to shear stress in the chamber, they must be seeded in a reverse manner onto the bottom of the insert membrane, as illustrated in Figure 8a. Among others, this method offers the advantage of improved optical access to the mucus layer and the mucus–cell interface and, thus, enhances the imaging quality in immunofluorescence experiments [54]. For a characterization of the epithelial barrier function of differently cultured HT29-MTX cell layers, we aimed at TEER measurements. In contrast to Caco-2 cells, which are the most commonly used model to mimic the intestinal barrier, HT29-MTX cells only form weak barriers, as indicated by low TEER values [55]. Beduneau et al. reported a time-dependent barrier formation of HT29-MTX cells developing from approximately 20 to 60 Ω∙cm^2^ from day 7 to 14 in static culture [17]. These values were comparable to our TEER-measurements, and also showed a time-dependent increase in the barrier strength, reaching 62.8 ± 2.2 Ω∙cm^2^ after three weeks of normal static culture (Figure 8b). Notably, an additional increase in the barrier function of HT29-MTX cell layers under reverse static conditions was observed, with a 2-fold increase in the TEER value up to 128.1 ± 5.6 Ω∙cm^2^ after three weeks of inverted culture. It is reported, with Caco-2 cells, that static, reverse, upside-down culture on inserts had a qualitatively similar effect on differentiation and cellular reorganization as the application of fluid flow [56]. Moreover, upon application of defined shear stress of 0.012 dyn∙cm^−2^ (12 × 10^−4^ Pa) to reversely cultured HT29-MTX cells, the maturation of the barrier was further accelerated compared to static cultures, resulting in significantly increased TEER values of 37.3 ± 2.1 Ω∙cm^2^ after one week followed by a more than 2-fold increase in the value, reaching 84.0 ± 3.6 Ω∙cm^2^ after only two weeks of dynamic culture (Figure 8b). At first glance, this increase seems to stagnate from two to three weeks of inverse dynamic culture (see dashed bar in Figure 8b). However, immunofluorescent stainings of the cytoskeleton via phalloidin revealed a similar formation of the villi-like structures detected on solid substrates when cultured under dynamic conditions (Figure 9a). The concomitant drastic increase in surface area under flow conditions thus resulted in TEER values that underestimated the reality. Therefore, we performed a correction of the value measured after three weeks of dynamic culture for the increased surface area (see Figure S13 for details). The resulting TEER value of 121.5 ± 8.1 Ω∙cm^2^ shows a clear increase after two weeks of dynamic culture and is in the same range as the one measured in static reverse conditions (Figure 8b).

To further investigate the epithelial barrier maturation, staining was conducted for the tight junction protein-1 (ZO-1), revealing a similar formation of tight junctions after three weeks of static reverse and dynamic reverse culture (Figure 9b). Beyond that, the amount of the main mucin MUC5AC produced by HT29-MTX cells was markedly increased after three weeks of culture under physiological flow, with comparable structural features as seen previously under dynamic conditions on solid substrates (Figure 9c). Additional control experiments again confirmed the biocompatibility of the 3D-printed resin, showing no influence of potential leaching on barrier function and mucus production (Figures S11 and S12). Overall, we were able to transfer the improved HT29-MTX-based mucus model, established in dynamic culture on solid substrates, to membranes as culture substrate, thus allowing additional measurements of the barrier function.

## 4. Conclusions

Reusable and sterilizable flow chambers were designed to apply physiologically relevant mechanical stress to intestinal HT29-MTX cells seeded on conventional microscopy slides or on membrane inserts. These conditions triggered the reorganization of the initial cellular monolayer, in the absence of cell-instructive gel matrices, towards a 3D structure that more closely resembles the physiological intestinal shape, with an increased surface area, via villi-like structure formation. Additionally, the required culture time for sufficient maturation towards a goblet-like phenotype was drastically reduced and a thick mucus layer could be detected, which was adherent to the cells. If singularized villi-like structures are desired in the model, a dynamic culture time of three weeks is recommended, while if only the produced mucus layer on the model is of interest, two weeks of dynamic culture are sufficient. Detailed analysis of the mucus revealed heavily branched networks of various mucins. We herein demonstrated the high impact of the application of physiological shear stresses of approximately 0.01 dyn∙cm^−2^ (1 × 10^−3^ Pa) on the cellular differentiation, mucus production, barrier formation and 3D reorganization of the cellular layer without further need for expensive culture supplements or equipment. In the future, by transferring this technique to the culture of intestinal monolayers derived from organoids or stem cells, their physiological behavior in terms of villi-like structure formation and especially mucus production could be enhanced. Therefore, the obtained results are an important contribution to the future development of advanced human mucus or improved intestinal models to mimic the intestinal barrier in vitro.

## Data Availability

Data are reported within the article and in the Supplementary Materials.

## Supplementary Materials

The following are available online at https://www.mdpi.com/article/10.3390/cells10082062/s1, Figure S1: Evaluation of flow properties in slide chambers, Figure S2: Surface shear stresses and velocity streamline profiles in slide chambers with varying channel heights, Figure S3: Setup for dynamic cell culture, Figure S4: Image processing and analysis to determine the native thickness of mucus produced by HT29-MTX cells, Figure S5: Colocalization of 3D self-organized and proliferative HT29-MTX cells, Figure S6: Confocal immunofluorescence images of HT29-MTX cells after static and dynamic culture at various time points, Figure S7: Influence of medium volume on cellular proliferation, Figure S8: Influence of medium volume on mucus production and intracellular ALP activity, Figure S9: Influence of medium volume on mucus structure, Figure S10: Biocompatibility of the 3D-printed resin, Figure S11: Influence of the 3D-printed resin on cellular barrier function, Figure S12: Influence of the 3D-printed resin on mucin expression and tight junction formation, Figure S13: Illustration of the surface area determination using ImageJ and Origin, Table S1: Calculated and simulated wall shear stress values, Table S2: Printing parameters for Dental SG Resin with LONGER Orange 30 printer, Table S3: Primary and secondary antibodies for immunofluorescent staining.
