# Supplementary material for: Physiological Shear Stress Enhances Differentiation, Mucus-Formation and Structural 3D Organization of Intestinal Epithelial Cells In Vitro
Source: Cells. 2021 Aug 12;10(8):2062. doi: 10.3390/cells10082062 (PMC8391940; doi:10.3390/cells10082062)
Supplement: Supplementary file 1 [file cells-10-02062-s001.zip › cells-1321827-supplementary.pdf]

Supplementary Information to the Manuscript "Physiological Shear Stress Enhances Differentiation, Mucus-Formation and Structural 3D Organization of Intestinal Epithelial Cells *in vitro*."

Marcus Lindner [1], Anna Laporte [2], Stephan Block [1], Laura Elomaa [1] and Marie Weinhart [1,2*]

[1] Institute of Chemistry and Biochemistry, Freie Universität Berlin, Berlin, Germany
[2] Institute of Physical Chemistry and Electrochemistry, Leibniz Universität Hannover, Hannover, Germany
* Correspondence: marie.weinhart@pci.uni-hannover.de; Tel.: +49 511 76214938

Outline

1. Chamber Design and Evaluation 2
2. 3D-Printing 3
3. Setup for Dynamic Cell Culture 4
4. Thickness-determination of the Adherent Mucus Layer 5
5. Colocalization of Proliferative and 3D Self-organized Cells. 6
6. Immunofluorescent Staining 6
7. Control Experiments – Medium Volume Adaption in Static Culture 7
8. Control Experiments – Material Leaching Assay to Assess Biocompatibility 9
9. Cellular Area Determination 11
10. References 12

1. Chamber Design and Evaluation

The range of applicable wall shear stresses was determined via mathematical approximation and validated by CFD simulation. Channel heights of the culture chamber and applied flow rates were varied from 0.15 to 6 mm and 2.84 to 14.2 mL/min, respectively (Table S1).

Table S1. Calculated and simulated wall shear stress values.

Channel Height [mm]	Homogenizer	Average surface shear stress [dyn/cm²] (CFD / calculated†)		
		2.84 mL/min (20 RPM) ‡	6.4 mL/min (45 RPM) ‡	14.2 mL/min (100 RPM) ‡
0.15	Threshold	4.6 / 5.9	10.4 / 13.2	23.1 / 29.7
0.35	Threshold	0.86 / 1.08	1.93 / 2.43	4.29 / 5.39
1.5	Threshold	0.055 / 0.059	0.124 / 0.132	0.277 / 0.294
6	Triangular	0.0039 / 0.0037	0.0088 / 0.0083	0.0196 / 0.0183

† mathematical approximation: $\tau = 6\mu Q w^{-1} h^{-2}$ with μ=0.93 cP

‡ valid for the OSPIN bioreactor system, using silicone tubings with an inner diameter of 1.6 mm.

CFD simulations were conducted to evaluate the fluid flow behavior within different channel geometries. Homogenous flow is enabled by a construction-dependent threshold for channel heights up to 1.5 mm, whereas a channel height of 6 mm necessitates the implementation of homogenizing structures (Figure S1).

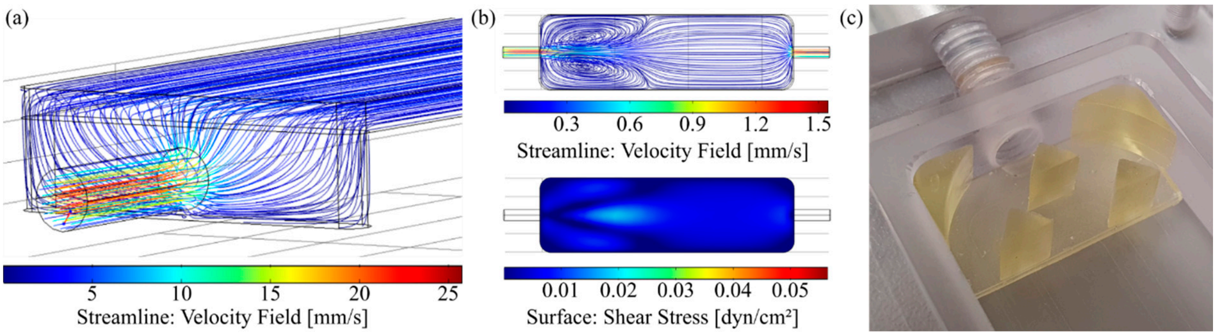

Figure S1. Evaluation of flow properties in slide chambers. (a) Fluid flow behaviour (velocity streamline profile) in a chamber with 1.5 mm channel height including a homogenizing threshold at a volume flow rate of 6.4 mL·min^{-1}. (b) Velocity streamline profile and surface shear stress in culture chambers without homogenizing structures with a channel height of 6 mm and a 6.4 mL·min^{-1} volume flow rate. (c) Photograph of a 3D-printed homogenizing insert (triangular) in a culture chamber.

Surface shear stress and velocity streamline profiles of all chambers at 6.4 mL min^{-1} (45 RPM) are shown in Figure S2.

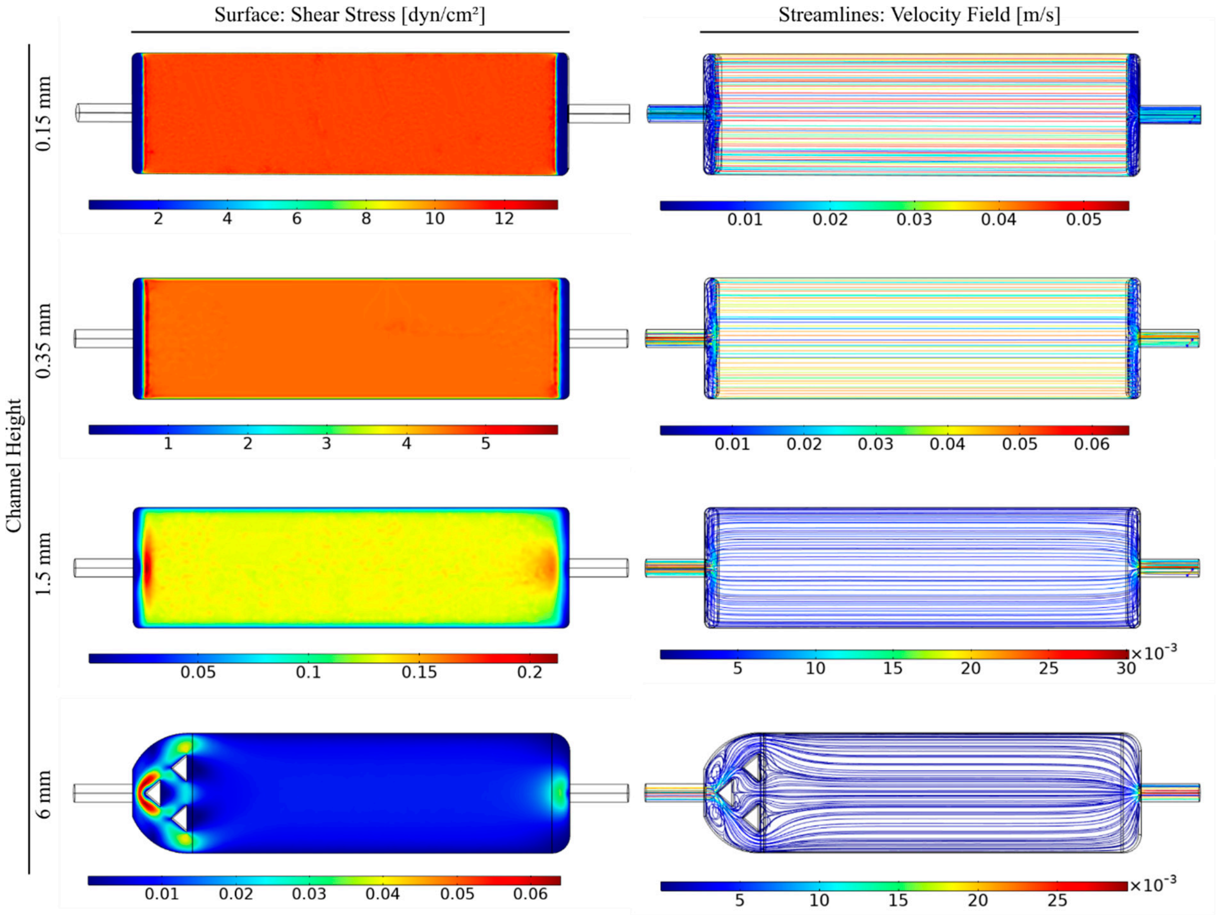

Figure S2. Surface shear stresses and velocity streamline profiles in slide chambers with varying channel heights from 0.15 to 6 mm including homogenizing structures at a volume flow rate of 6.4 mL·min^{-1}.

2. 3D-Printing

Homogenizing structures and chambers for dynamic insert culture were CAD-modelled with *Rhinoceros* (Version 5.0, Robert McNeel & Associates, Seattle, WA/USA) and exported as an stl-file. We used a LONGER Orange 30 printer (Shenzhen, China) and Formlabs Dental SG resin (Somerville, MA/USA), which is classified as biocompatible according to EN-ISO 10993-1:2009/AC:2010 to print the models. The stl-file was imported into the printer's own slicing software *LongerWare* v1.32. The models were manually aligned in x- and y-position and tilted, if necessary. Supporting structures were added with default specifications and the model was sliced with a layer thickness of 50 µm. The resin-specific printing parameters can be found in Table S2. After exporting the lgs-file and printing, the parts were removed from the building platform and washed twice in 99% isopropyl alcohol or ethanol, followed by a post-curing step in a self-made chamber using a 30 W UV-A LED lamp (λ = 385-400 nm) for 60 seconds each (top & bottom). The models were finally washed excessively with warm tap water and extracted in deionized water at 37 °C for at least 24 h.

Table S2. Printing parameters for Dental SG Resin with LONGER Orange 30 printer.

Parameter		Value
Burn-In Time [ms]	:	60000
Burn-In Range [mm]	:	0.3
Curing Time [ms]	:	7500
Stable Time [ms]	:	1000
Lift Distance 1 [mm]	:	2
Lift Speed 1 [mm/min]	:	48
Lift Distance 2 [mm]	:	2
Lift Speed 2 [mm/min]	:	300

3. Setup for Dynamic Cell Culture

The dynamic culture of cells on solid surfaces was performed using OSPIN's bioreactor system. A photo of the setup without the closing lid is shown in Figure S3a. Peristaltic pumps provide perfusion of the circuit and enable an automatic media exchange. The pH-value is monitored on-line and controlled via passive diffusion of CO_2 through a silicone tubing in the gassing unit. With the system closed, a temperature of 37 °C is maintained. A simpler system was used for cultivation of cells on cell culture inserts, with a peristaltic pump directly operated in a cell culture incubator under standard conditions (37 °C, 5% CO_2), shown in Figure S3b. A bubble trap and four culture chambers were mounted in line as a circuit.

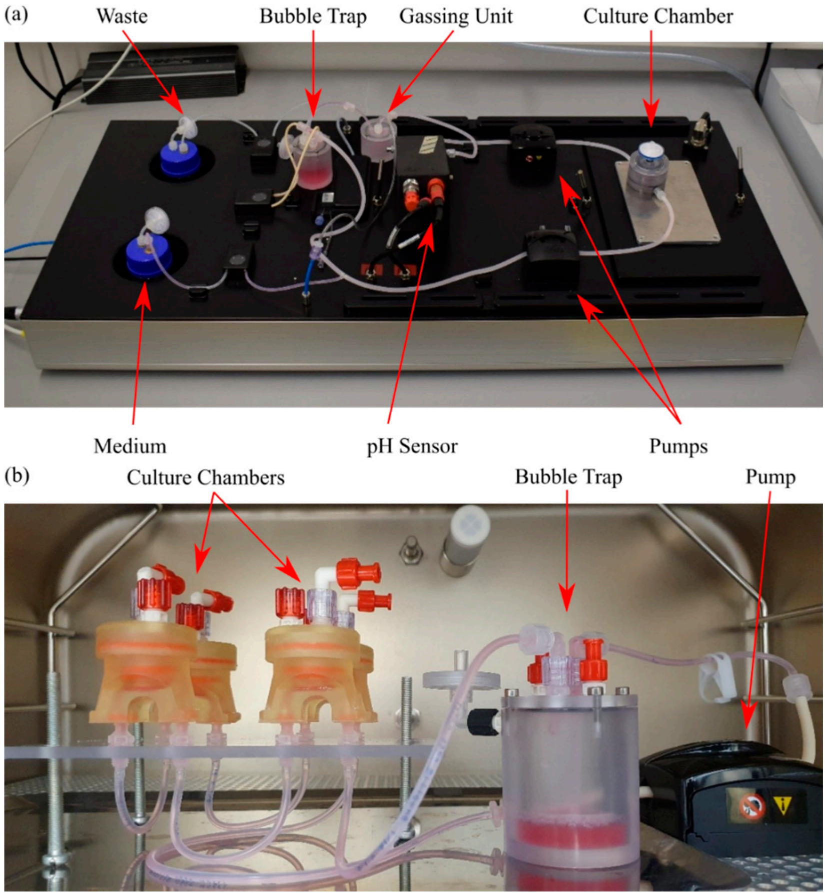

Figure S3. Setup for dynamic cell culture. A labeled photograph of (a) the bioreactor system and (b) a peristaltic pump, operated in a cell culture incubator, including the circuits for the dynamic culture of cells on solid substrates and cell culture inserts, respectively.

4. Thickness-determination of the Adherent Mucus Layer

To determine mucus thickness, unfixed samples were incubated with fluorescence-labelled particles as well as Hoechst and subsequently analyzed using confocal microscopy [1, 2]. Multiple frame averaging (4x) is essential during data acquisition. The raw data (Z-stacks, 8-bit grayscale) is converted into binary images via manual thresholding in *ImageJ*. Frame averaging allows the exclusion of unbound particles via their reduced intensity. Particle analysis is then performed using *BoneJ* plugin, applying a size filter of 500-2500 and 5-150 µm³ for nuclei and beads, respectively (Figure S4a). The obtained coordinates (X, Y and Z position) were verified (exemplified in Figure S4b) and allotted to groups in a 10x10 heatmap with *Origin* to prevent areas with high bead-density from falsifying the results (Figure S4c). Finally, corresponding clustered data points were subtracted from each other (beads minus nuclei). The slight overestimation of thickness due to the distance from nucleus center to cell surface was deliberately ignored since nuclei had an average radius of approximately 6 ± 2 µm and were thus within the margin of error.

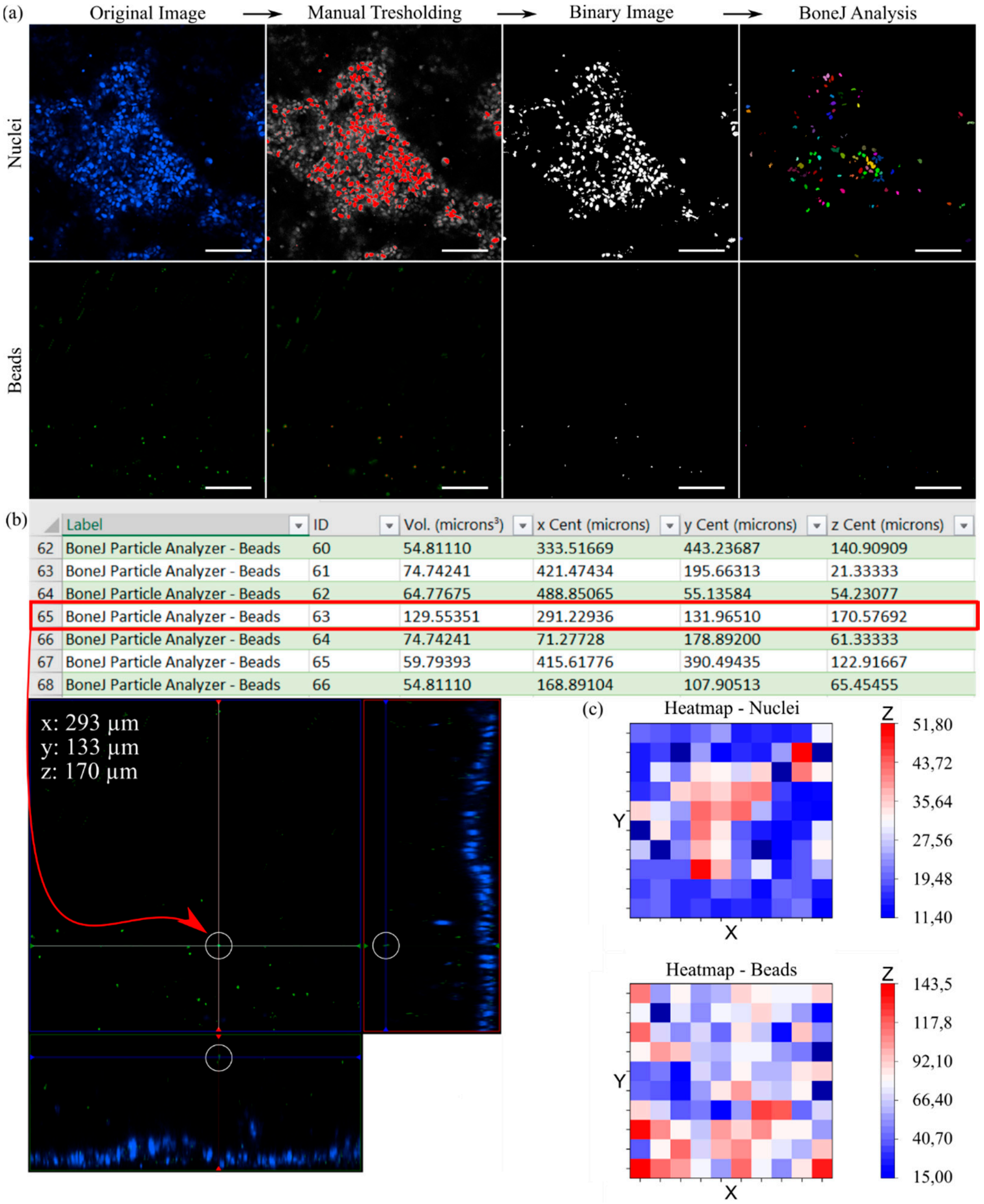

Figure S4. Image processing and analysis to determine the native thickness of mucus produced by HT29-MTX cells. (a) The raw data is converted into a binary image via manual thresholding and analyzed via *BoneJ*'s particle analyzer. The results were verified, as illustrated exemplary in (b). All data points were plotted in *Origin* as a 10x10 matrix and finally subtracted (beads – nuclei; (c)). (scale bar 100 µm)

5. Colocalization of Proliferative and 3D Self-organized Cells.

As the confocal microscope used for acquisition of fluorescence images shown in the main manuscript is not equipped with a camera for brightfield imaging, we examined HT29-MTX cells after 3 weeks of dynamic culture on a normal inverted epifluorescence microscope, after staining with Hoechst and EdU for nuclei and proliferative cells, respectively. The dark areas, which are pronounced after dynamic culture, show a clear colocalization with proliferating cells, illustrated by the merged images in Figure S5.

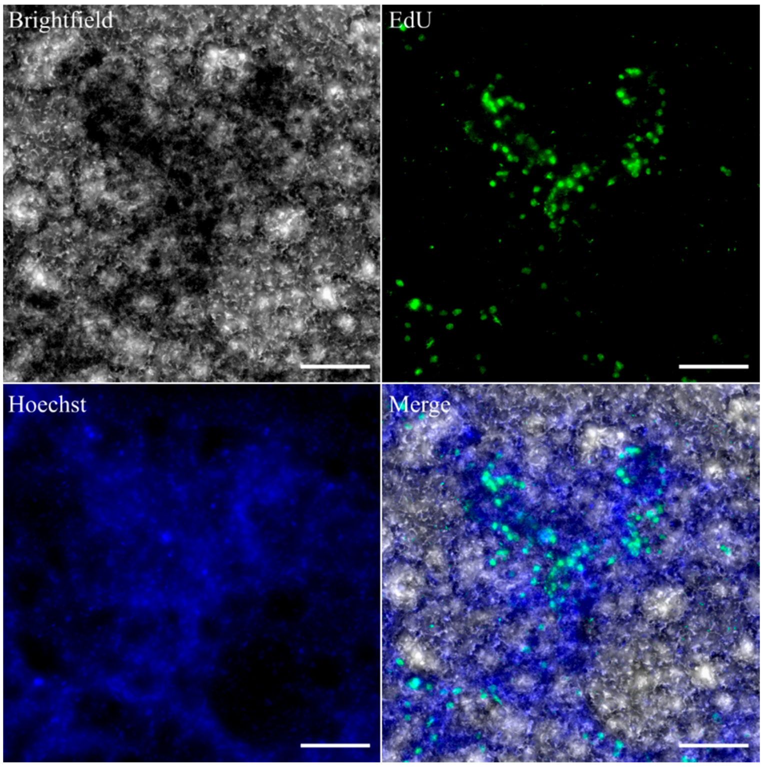

Figure S5. Colocalization of 3D self-organized (dark areas, brightfield) and proliferative HT29-MTX cells (green, EdU), obtained via brightfield and epifluorescence microscopy, respectively. Nuclei were counterstained with Hoechst 33342 (blue). (scale bar 100 µm)

6. Immunofluorescent Staining

For immunofluorescent staining of selected mucins and ZO-1, the following antibodies and dilutions were used.

Table S3. Primary and secondary antibodies for immunofluorescent staining.

Target	Species	Dilution	2nd Antibody (1:500)
MUC1	Rabbit (polyclonal)	1:200	Goat-α-rabbit (H+L) Alexa-488®
MUC2	Mouse (monoclonal)	1:100	Goat-α-mouse (IgG1) Alexa-488® (counterstained with Phalloidin-Atto647®#)
MUC5AC	Mouse (monoclonal)	1:250	Goat-α-mouse (IgG1) Alexa-488®
MUC5B	Rabbit (polyclonal)	1:100	Goat-α-rabbit (H+L) Alexa-488®
ZO-1	Rabbit (polyclonal)	1:250	Goat-α-rabbit (H+L) Alexa-647®
Co-Staining			
MUC5AC	Mouse (monoclonal)	1:250	Goat-α-mouse (IgG1) Alexa-647®
MUC5B	Rabbit (polyclonal)	1:100	Goat-α-rabbit (H+L) Alexa-488®

diluted 1:200 in PBS, incubation for 1 h at room temperature

Confocal images of immunofluorescent stainings of HT29-MTX cells after dynamic and static culture for up to three weeks on solid substrates are shown in Figure S6.

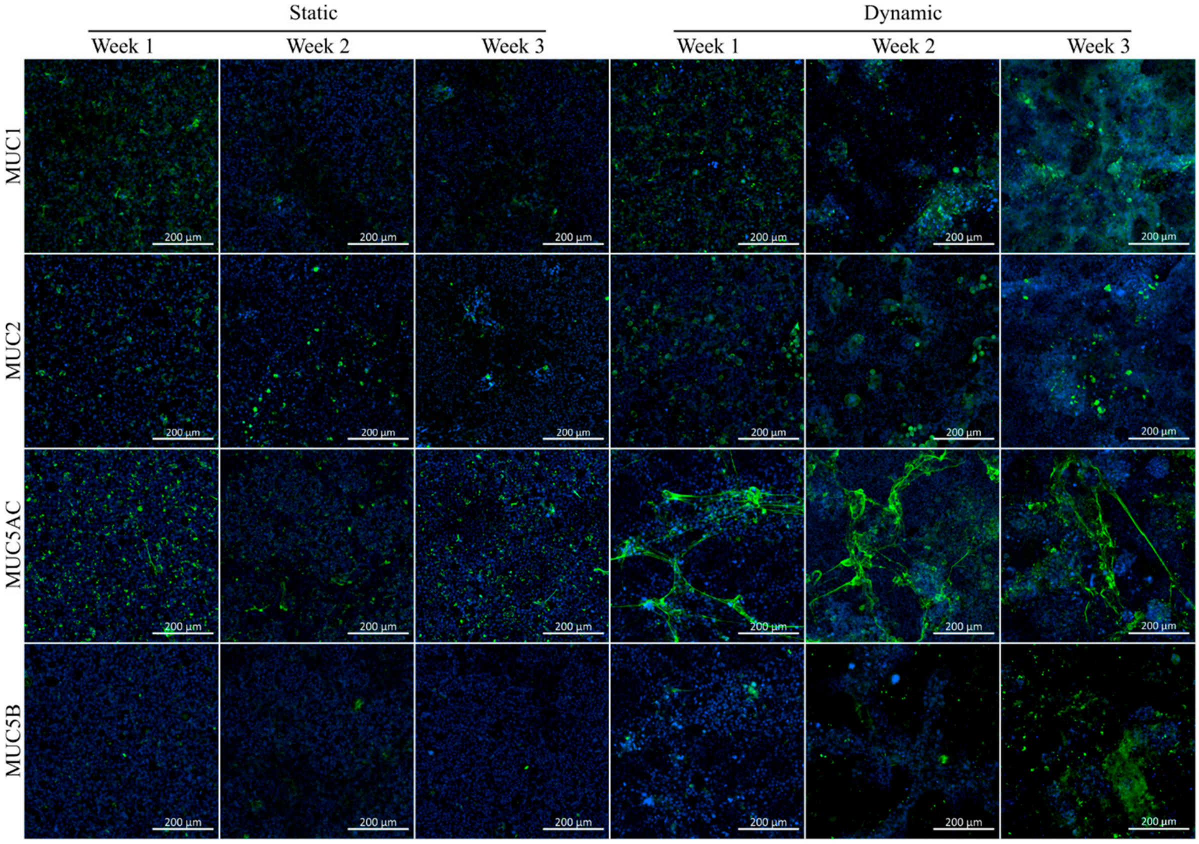

Figure S6. Confocal immunofluorescence images of HT29-MTX cells after static and dynamic culture at various time points stained with antibodies for the membrane-bound mucin MUC1 and secreted mucins MUC2, MUC5AC and MUC5B (green). Nuclei were counterstained with Hoechst 33342 (blue).

7. Control Experiments – Medium Volume Adaption in Static Culture

Since the bioreactor circuit contains a higher volume of cell culture medium compared to static culture on solid substrates, control experiments were performed to ensure that the observed effects were caused by the shear stress in dynamic culture only and were not related to the higher medium volume. Cells were either cultured under standard conditions (Static Culture), dynamic conditions (Dynamic Culture) or static conditions with similar ratio of medium volume to cell seeding area as in the dynamic culture (Medium control). Changes in proliferation (Figure S7), mucus production (Figure S8a), ALP activity (Figure S8b) and mucus structure (Figure S9) induced by higher medium volume were not comparable to the ones seen in dynamic culture. The cell proliferation was even weaker than in conventional static culture after three weeks and mucin amounts tended to be much lower due to the higher dilution of mucins under static conditions with high medium volume [3].

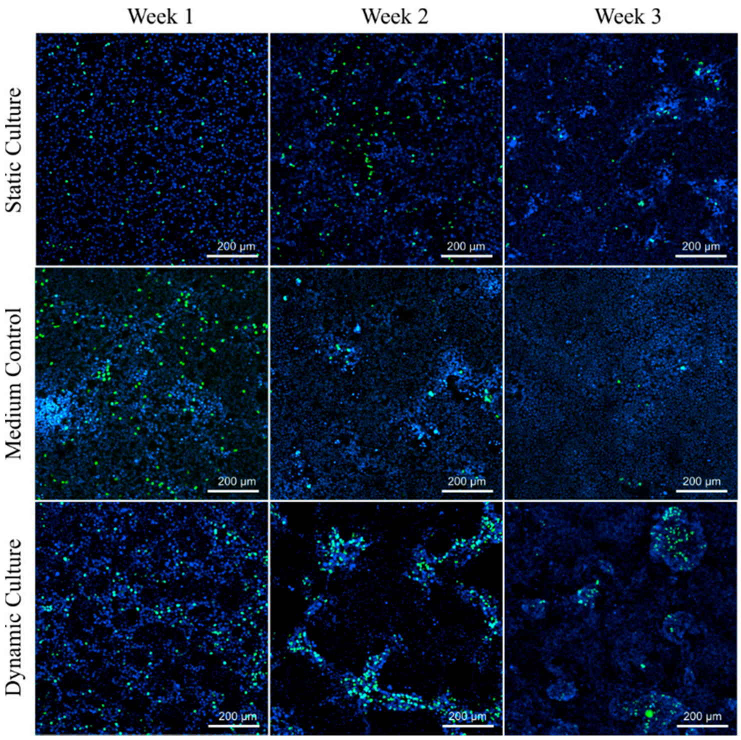

Figure S7. Influence of medium volume on cellular proliferation. HT29-MTX cells were cultured for up to three weeks under conventional conditions (Static Culture), dynamic conditions (Dynamic Culture), and static conditions with media volumes comparable to dynamic culture (Medium Control). n=2

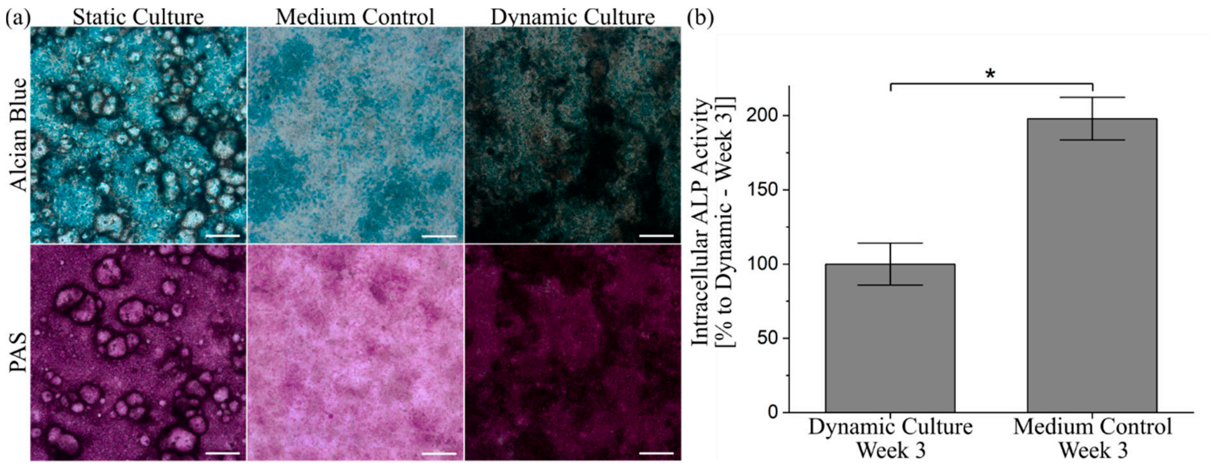

Figure S8. Influence of medium volume on mucus production and intracellular ALP activity. (a) Alcian blue and PAS staining of HT29-MTX after three weeks of culture under conventional conditions (Static Culture), dynamic conditions (Dynamic Culture), and static conditions with media volumes comparable to dynamic culture (Medium Control) n=2. (b) Intracellular ALP activity after three weeks of culture under dynamic conditions and static conditions with media volumes comparable to dynamic culture (Medium Control). Data presented as mean ± SEM with respect to week 3 under dynamic conditions. n=4-8

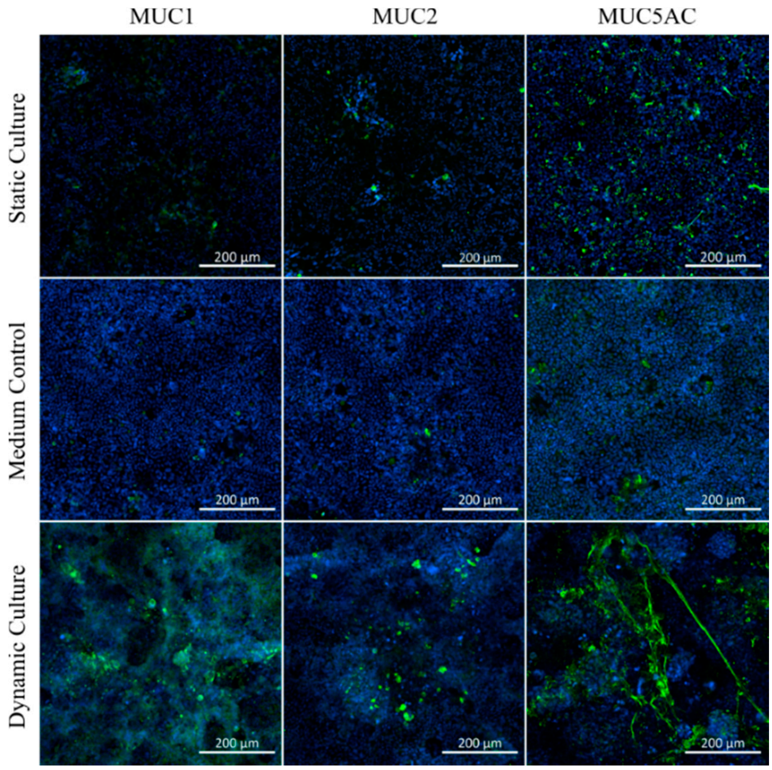

Figure S9. Influence of medium volume on mucus structure determined by immunofluorescent staining with antibodies for MUC1, MUC2 and MUC5AC. HT29-MTX cells were cultured for three weeks under conventional conditions (Static Culture), dynamic conditions (Dynamic Culture), and static conditions with media volumes comparable to dynamic culture (Medium Control). n=2

8. Control Experiments – Material Leaching Assay to Assess Biocompatibility

To evaluate the biocompatibility of the printed resin, we statically cultured HT29-MTX cells on inserts for three weeks either conventionally in 12-well plates (Static Culture) or in 3D-printed wells (Leaching Control), with similar ratio of medium volume to contact area to the material as in the chamber for dynamic culture. Subsequently a live/dead staining using fluorescein diacetate and propidium iodide was conducted and samples were analyzed via confocal microscopy, shown in Figure S10.

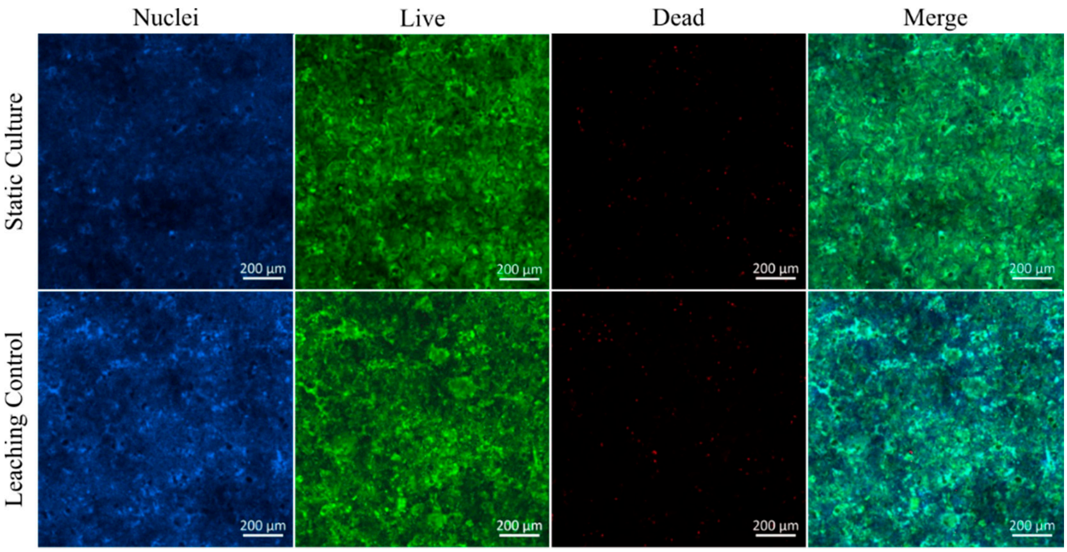

Figure S10. Biocompatibility of the 3D-printed resin. HT29-MTX cells were cultured for three weeks in conventional (Static Culture) and 3D-printed (Leaching Control) wells and subsequently incubated with fluorescein diacetate (green) and propidium iodide (red) to identify living and dead cells, respectively. Nuclei were counterstained with Hoechst 33342 (blue). n=2

The same culture setup was used to exclude an influence of the crosslinked resin on barrier function, mucus production and tight junction formation, as shown in Figures S11 and S12.

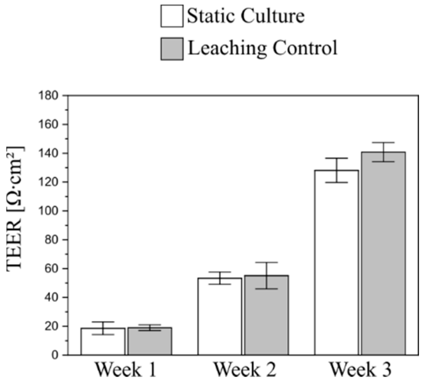

Figure S11. Influence of the 3D-printed resin on cellular barrier function. HT29-MTX cells were cultured for three weeks in conventional (Static Culture) and 3D-printed (Leaching Control) wells. TEER values were measured every 7 days. Data presented as mean ± SEM. n=4-12

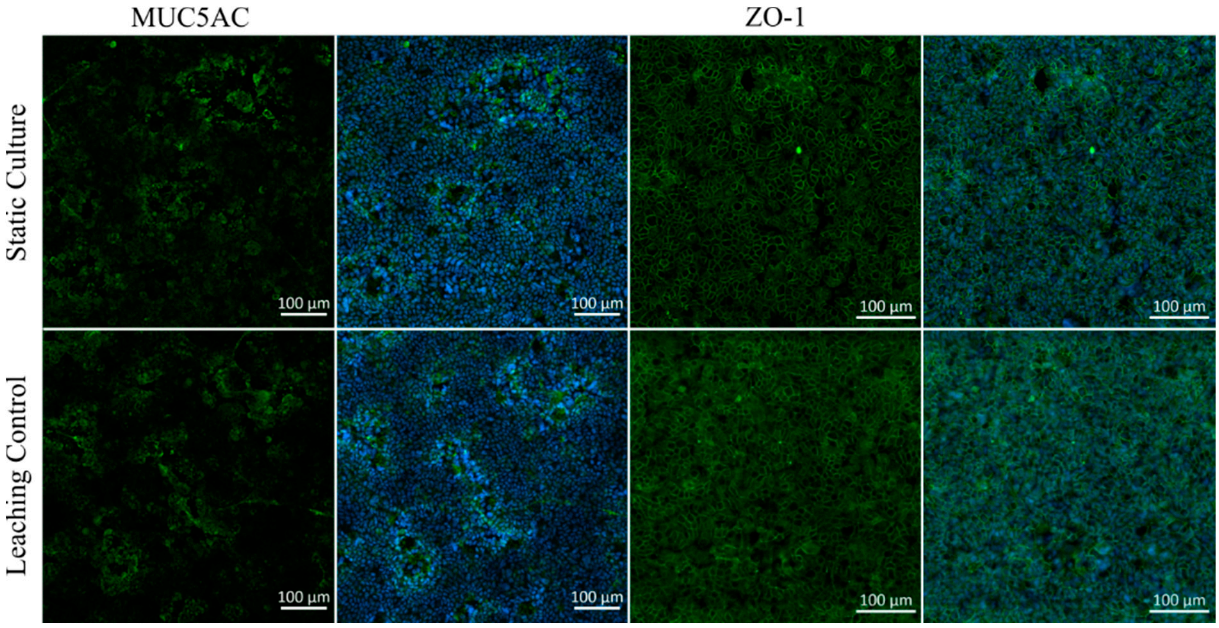

Figure S12. Influence of the 3D-printed resin on mucin expression and tight junction formation. HT29-MTX cells were cultured for three weeks in conventional (Static Culture) and 3D-printed (Leaching Control) wells and stained for the secreted mucin MUC5AC and for ZO-1 as an integral part of tight junctions. n=2

9. Cellular Area Determination

To determine the respective surface area of cells cultured statically and dynamically on membrane inserts, we analyzed confocal images of cell layers stained using phalloidin after three weeks under both culture conditions with *ImageJ* and *Origin*. To evaluate the method, a hemisphere with a radius of 125 µm on a 500 x 500 µm flat surface was CAD-modelled, sliced with a layer thickness of 5 µm and imported in *ImageJ*. The topography was computed with *TopoJ*-plugin, resulting in a 2-dimensional image, which was subsequently smoothed by applying a gaussian filter. The image was exported as a text file, imported into *Origin* and 3D-plotted. The surface area of the plot can be calculated via the *xyzarea* command and resulted in a reasonable area of 0.295 mm², with a deviation of only 1.3% to the theoretical value: base area (0.25 mm²) minus circular area (0.049 mm²) plus area of hemisphere (0.098 mm²). Cellular surface areas were determined identically (Figure S13).

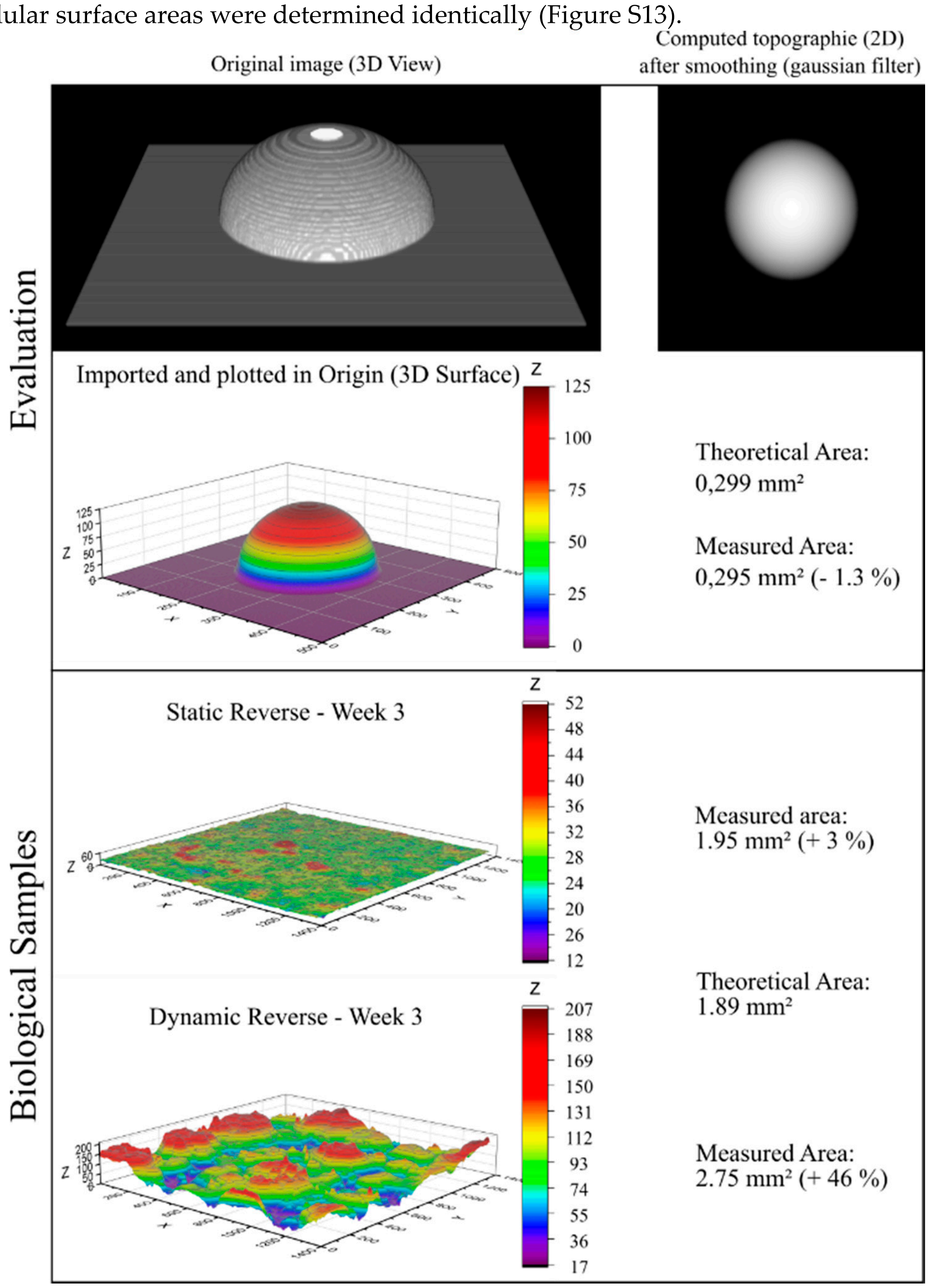

Figure S13. Illustration of the surface area determination using *ImageJ* and *Origin*. For the evaluation, a 3D model of a hemisphere was CAD-modelled, sliced and imported into *ImageJ*. The topography was computed with the *TopoJ*-plugin and a gaussian filter was applied to smoothen the surface. The file was exported as a text file, imported into *Origin*, plotted as a 3D-surface and the surface area was determined. The same procedure was performed for confocal images of cells stained with phalloidin.

10. References

1. Johansson, M.E.; Gustafsson, J.K.; Holmen-Larsson, J.; Jabbar, K.S.; Xia, L.; Xu, H.; Ghishan, F.K.; Carvalho, F.A.; Gewirtz, A.T.; Sjovall, H.; et al. Bacteria penetrate the normally impenetrable inner colon mucus layer in both murine colitis models and patients with ulcerative colitis. *Gut* **2014**, *63*, 281-291; DOI:10.1136/gutjnl-2012-303207.
2. Furter, M.; Sellin, M.E.; Hansson, G.C.; Hardt, W.D. Mucus Architecture and Near-Surface Swimming Affect Distinct Salmonella Typhimurium Infection Patterns along the Murine Intestinal Tract. *Cell Rep.* **2019**, *27*, 2665-2678 e2663; DOI:10.1016/j.celrep.2019.04.106.
3. Wang, Y.; Kim, R.; Sims, C.E.; Allbritton, N.L. Building a Thick Mucus Hydrogel Layer to Improve the Physiological Relevance of In Vitro Primary Colonic Epithelial Models. *Cell. Mol. Gastroenterol. Hepatol.* **2019**, *8*, 653-655 e655; DOI:10.1016/j.jcmgh.2019.07.009.
